# Microbiomic association between the saliva and salivary stone in patients with sialolithiasis

**DOI:** 10.1038/s41598-024-59546-x

**Published:** 2024-04-22

**Authors:** Jiwon Park, Soo Yeon Jung, Ha Yeong Kim, Kyeong Eun Lee, Yu Jin Go, Han Su Kim, Seo-yoon Yoon, Cheol-O Kwon, Yoon Shin Park

**Affiliations:** 1 Department of Biological Sciences and Biotechnology, School of Biological Sciences, College of Natural Sciences, Chungbuk National University, Cheongju, 28644 Republic of Korea; 2 Department of Otorhinolaryngology-Head and Neck Surgery, College of Medicine, Ewha Womans University, Seoul, 07865 Republic of Korea; 3 MD Healthcare Inc., Seoul, 03923 Republic of Korea; 4 Department of Otorhinolaryngology-Head and Neck Surgery, College of Medicine, Ewha Womans University, 1071 Anyangcheon-ro, Yangcheon-gu, Seoul, 07985 Republic of Korea

**Keywords:** Salivary stone, Saliva, Sialolithiasis, Oral microbiome, Oral diseases, Biomarkers, Immunopathogenesis

## Abstract

Salivary stones, known as sialoliths, form within the salivary ducts due to abnormal salivary composition and cause painful symptoms, for which surgical removal is the primary treatment. This study explored the role of the salivary microbial communities in the formation of sialoliths. We conducted a comparative analysis of microbial communities present in the saliva and salivary stones, and sequenced the 16S rRNA gene in samples obtained from patients with sialoliths and from healthy individuals. Although the diversity in the saliva was high, the essential features of the microbial environment in sialoliths were low diversity and evenness. The association of microbial abundance between stones and saliva revealed a positive correlation between *Peptostreptococcus* and *Porphyromonas*, and a negative correlation for *Pseudomonas* in saliva. The functional potential differences between saliva and stones Bacterial chemotaxis and the citrate cycle were negatively correlated with most genera found in salivary stone samples. However, the functions required for organic compound degradation did not differ between the saliva samples. Although some microbes were shared between the sialoliths and saliva, their compositions differed significantly. Our study presents a novel comparison between salivary stones and salivary microbiomes, suggesting potential preventive strategies against sialolithiasis.

## Introduction

Sialolithiasis is a common disease characterized by the formation of stones in salivary glands and ducts, causing symptoms related to obstruction and inflammation, such as pain and glandular swelling. It occurs in 0.1–1% of the population^1^. If a stone causes symptoms, surgical removal is the only available treatment option. Palpable stones near the orifice are easily removed transorally under local anesthesia. However, unpalpable stones located near the submandibular gland require surgery under general anesthesia^2,3^. Sialendoscopy and intraoperative ultrasound have been recently used to determine the precise location of salivary stones; however, these two methods require general anesthesia surgery^4,5^. In addition, recurrent sialolithiasis has also been reported after stone removal^6^. To avoid stone formation, warm compression, increasing fluid intake, and massaging of the salivary glands, which can stimulate saliva production and possibly flush the stones, are recommended^1^. However, there are currently no preventive treatments that inhibit stone formation. A comprehensive understanding of the precise etiology of stone formation is required to develop a preventive therapeutic approach^7,8^.

The formation of calcified deposits within the salivary glands causes sialolithiasis; however, little is known about the pathophysiology of salivary gland stones. A decreased salivary flow is a well-established factor. The main causes of sialolithiasis are salivary retention and composition. Comparatively, the concentration of calcium in the saliva of patients with sialolithiasis is higher than that in healthy individuals, while the concentration of crystallization-inhibiting phytate is lower^9^. Changes in the oral environment, including pH, hydration, inflammation, and bacterial composition and distribution, may facilitate stone formation^10^. Among these factors, bacteria has been reported to play a crucial role in the deposition of calcium during sialolith formation, acting as crystallization nuclei^1–3^. Moreover, previous studies analyzing sialoliths have detected the presence of bacteria and of a biofilm, suggesting an association between bacteria and sialoliths. Several studies have investigated the bacterial species present in salivary stones. Since salivary stones form in the environment of saliva secreted by salivary glands, it is presumed that the composition of the salivary stone microbiome would be influenced by the microbiome composition of saliva. However, to the best of our knowledge, no study has identified and compared the core microbial community profiles of saliva in patients with sialolithiasis^11–14^.

In this study, we focused on the distribution and correlation of the microbiome in the salivary stones and saliva of healthy individuals and patients with sialolithiasis. First, we investigated whether the microbial distribution in the saliva and stones was associated. Next, we investigated differences in the microbial environment in the saliva of people with and without sialoliths to determine which microbes present in the saliva are associated with sialoliths.

The association between functional changes in the microbes and alterations in the microbiome found in the saliva and in salivary stones was analyzed. To the best of our knowledge, this is the first comparative analysis of the microbiome found in the saliva and in salivary stones. Our findings could aid in the identification of the causes of salivary stone formation and in the development of proactive treatment strategies.

## Results

### Demographic and laboratory findings of the patients and characteristics of the stones

The general characteristics and biochemical indices of the 27 participants in the control group and 27 participants in the salivary stone group are presented along with the corresponding *P*-values in Table 1. Mean anthropometric measurements for all participants were: age, 41.00 ± 13.92 years; height, 166.97 ± 8.31 cm; body weight, 66.67 ± 12.79 kg; and body mass index (BMI), 23.84 ± 3.79 kg/m^2^. Those specific for the control group were: age, 39.81 ± 12.12 years; height, 166.49 ± 7.02 cm; body weight, 65.39 ± 12.62 kg, and BMI, 23.46 ± 3.38 kg/m^2^; whereas those for the salivary stone group were: age, 42.19 ± 15.66 years; height, 167.45 ± 9.54 cm; body weight, 67.94 ± 13.07 kg; and BMI, 24.22 ± 4.18 kg/m^2^. No significant differences in anthropometric measurements or biochemical indices were observed between the two groups. Only oral health-associated behavior, namely, the number of tooth brushings per day, demonstrated a significant difference between the two groups. The number of stones ranged from 1 to 4, and the average size was 8.07 ± 3.65 mm in length, 5.65 ± 2.18 mm in width, and 7.41 ± 3.20 mm in height, with a total volume of 190.46 ± 222.68 mm^3^ (Supplementary Table S1).

**Table 1:** General characteristics and biochemical indices of the participants.

Parameters		Total (Mean ± S.D.)			Healthy individuals (Mean ± S.D.)			Sialolithiasis patients (Mean ± S.D.)			P value
Anthropometric measurement	Male		22			10			12		
	Female		32			17			15		
	Age (years)	41	±	13.92	39.81	±	12.12	42.19	±	15.66	0.863
	Height (cm)	166.97	±	8.31	166.49	±	7.02	167.45	±	9.54	0.667
	Weight (kg)	66.67	±	12.79	65.39	±	12.62	67.94	±	13.07	0.446
	BMI (kg/m^2^)	23.84	±	3.79	23.46	±	3.38	24.22	±	4.18	0.47
Biochemical Indices	WBC	6.43	±	1.65	6.54	±	1.53	6.34	±	1.76	0.644
	N	59.39	±	9.32	62	±	10.41	57.26	±	7.9	0.076
	Lympho	31.43	±	8.51	29.63	±	9.15	32.89	±	7.82	0.186
	Mono	6.68	±	1.78	6.35	±	1.73	6.95	±	1.81	0.243
	Eosino	1.89	±	1.43	1.52	±	0.87	2.2	±	1.72	0.277
	Hb	14.01	±	1.42	13.99	±	1.34	14.03	±	1.52	0.926
	PLT	264.92	±	69.85	272.14	±	66.85	259.04	±	72.93	0.527
	Glu	94.9	±	9.9	92.73	±	6.75	99.23	±	13.6	0.18
	BUN	13.24	±	3.94	12.68	±	3.77	13.77	±	4.1	0.329
	Cr	0.75	±	0.15	0.77	±	0.17	0.72	±	0.12	0.303
	T.Ca	9.65	±	0.29	9.5	±	0.19	9.68	±	0.3	0.174
	P	3.6	±	0.54	3.36	±	0.7	3.64	±	0.51	0.291
Oral health behavior	Alcohol drinking (cups per week)	4.032	±	7.56	3.713	±	6.28	4.35	±	8.757	0.759
	Alcohol drinking (frequency per week)	0.954	±	1.23	1.176	±	1.358	0.73	±	1.07	0.188
	Water intake (cups per day)	4.361	±	2.4656	4.09	±	0.43	4.63	±	0.52	0.673
	Toothbrush (frequency per day)	2.648	±	0.6773	2.96	±	0.06	2.33	±	0.15	0.000*
	Dental floss (frequency per day)	0.370	±	0.4874	0.407	±	0.5	0.33	±	0.48	0.58

Anthropometric measurements and biochemical indices are represented as the mean ± standard deviation. Differences between the control and stone groups were analyzed using an unpaired *t*-test and one-way analysis of variance, followed by Tukey’s post-hoc test.

### Total number of identified and classified microbes by category

To compare the microbiomes among the control saliva, stone saliva, and stone groups, 16S rRNA amplicon sequencing was conducted, and 80 samples were processed. The compositions of the microbiota in both saliva groups and stone group were determined at the taxonomic level (Supplementary Table S2). In the control saliva group, 8 phyla, 13 classes, 29 orders, 44 families, 64 genera, and 158 species were annotated, while 10 phyla, 15 classes, 32 orders, 47 families, 73 genera, and 170 species were annotated in the stone saliva group. In the stone group, 7 phyla, 12 classes, 27 orders, 42 families, 49 genera, and 76 species were annotated. At the genus level, the control and stone saliva groups exhibited a greater number of genera than the stone group.

### Comparison between alpha and beta diversities

**Figure 1:**
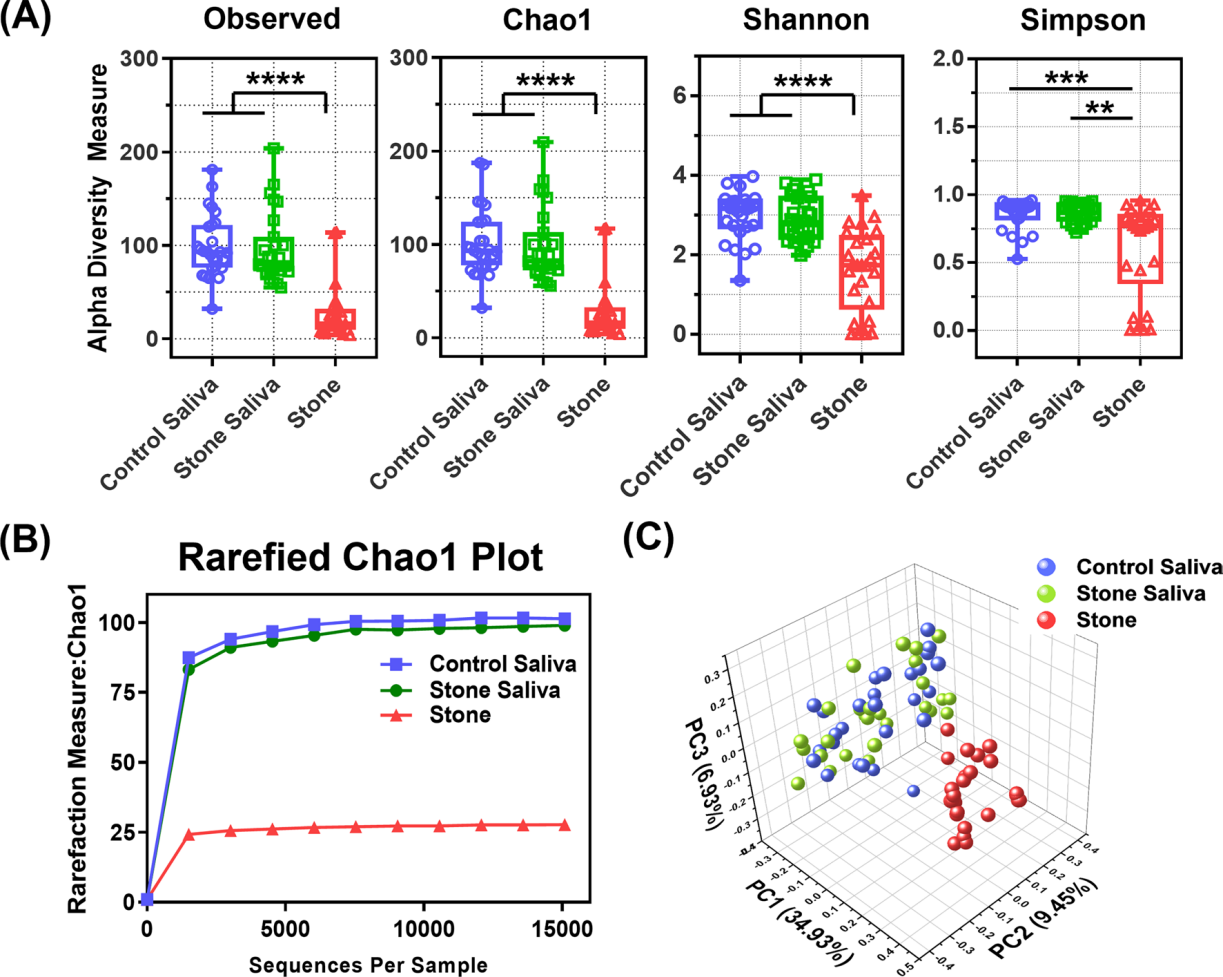
Comparison with alpha- and beta-diversity for saliva and stone samples. (**A**) Rarefaction curve (**B**) Beta diversity-based principal component analysis (PCA) plots of Bray**–**Curtis-computed distances. (**C**) Box plots for alpha-diversity based on observed OTUs, Chao1, Shannon, and Simpson indices. Blue, green, and red represent saliva from healthy individuals, saliva from patients, and salivary stones, respectively. Wilcoxon rank-sum test for differences in alpha-diversity between groups. (**P < 0.01, ***P < 0.001, ****P < 0.0001).

Alpha diversity assessment, which indicates species abundance within each group, and beta diversity assessment based on principal component analysis (PCA) were performed. No significant differences were observed in the operational taxonomic units (OTUs), Chao1, and Shannon indices between the saliva groups, whereas there was a significant difference between the saliva and stone groups (*P* < 0.0001), with stones showing less diversity than saliva (Fig. 1A). The Simpson index showed no significant difference between the saliva groups; however, there was a significant difference between the control saliva and stone groups (*P* = 0.0007), and between the stone saliva and stone groups (*P* = 0.003). Rarefaction curves are shown in Fig. 1B. PCA was used to compare the diversity between the groups, and the saliva and stone groups were distinguished based on the PC1 score (Fig. 1C). Statistical differences between the groups were assessed for significance using a one-way analysis of similarities (ANOSIM) of the microbial communities (R = 0.3575, *P* = 0.0001). The ANOSIM revealed significant differences between the control and stone saliva groups (R = 0.0211, *P* > 0.05), between the control saliva group and stone group (R = 0.5148, *P* = 0.0001), and between the stone saliva and stone groups (R = 0.5207, *P* = 0.0001).

### Microbial composition at the phylum and genus level

**Figure 2:**
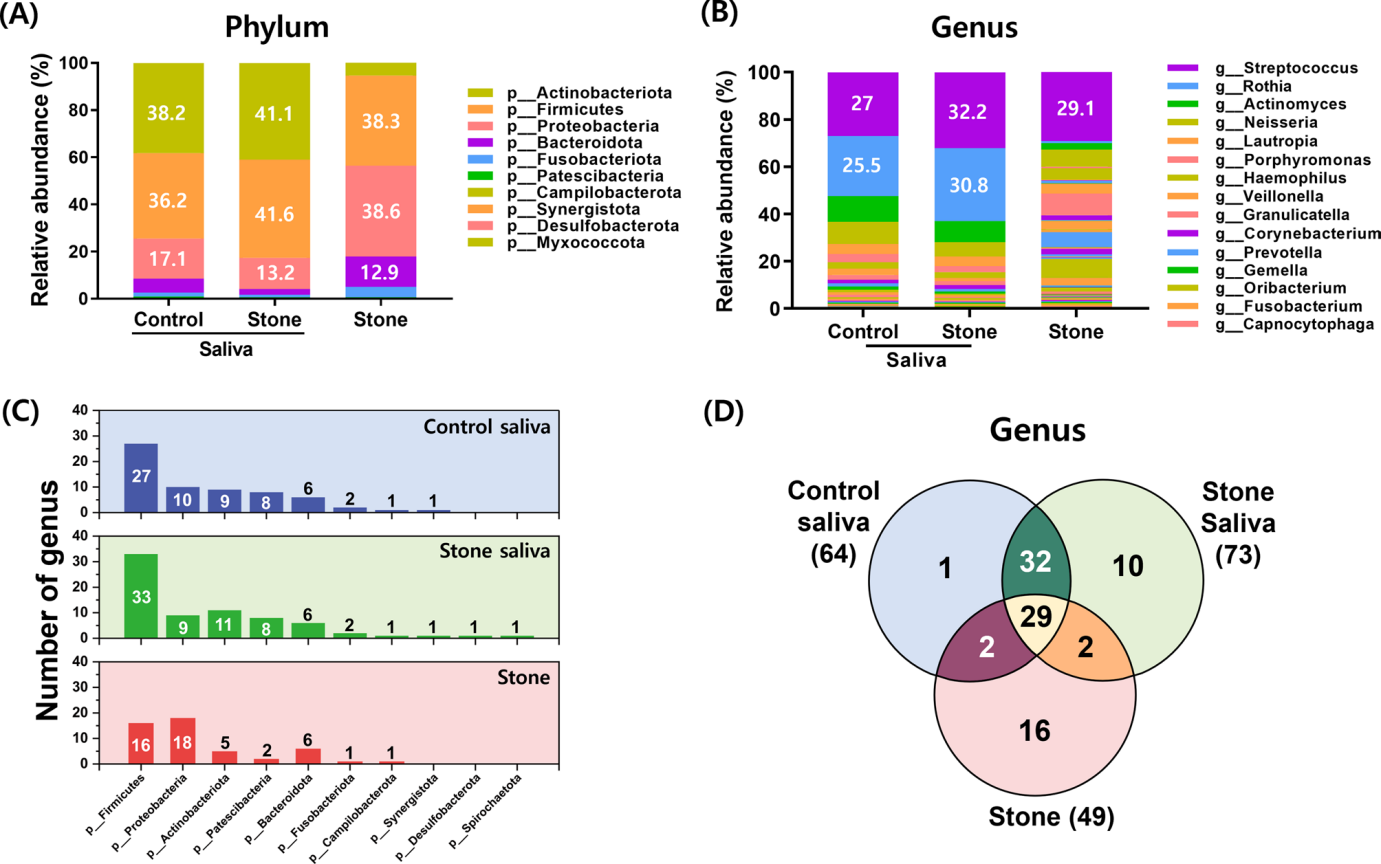
Comparison of the microbial composition of the saliva and salivary stones. Relative abundances at the (**A**) phylum and (**B**) genus levels in saliva from healthy individuals, saliva from patinets, and salivary stones. The relative abundances of the microbial communities were based on the dominant phyla and genera. (**C**) Number of genera in the three groups at the phylum level. (**D**) Venn diagram showing the overall overlap among the three groups.

We identified the microbiome compositions of the saliva and salivary stone samples based on the average relative abundances at the phylum and genus levels. The main phyla in the saliva samples were Actinobacteria, Firmicutes, Proteobacteria, and Bacteroidetes (Fig. 2A). These four dominant phyla accounted for more than 90% of total bacteria, and there were no significant differences between saliva samples from patients and from healthy individuals at the phylum level. In contrast, the main phyla in the salivary stone samples were Proteobacteria, Firmicutes, Bacteroidetes, and Actinobacteria. The abundance of Proteobacteria and Actinobacteria was significantly different between the saliva groups and the salivary stone group.

At the genus level, *Streptococcus* (27.02% in saliva samples from healthy individuals, 32.22% in samples from patients) and *Rothia* (25.53% in saliva samples from healthy individuals; 30.77% in samples from patients) accounted for more than half of the total abundance in the saliva samples (Fig. 2B, Table 2). *Streptococcus* showed significantly dominant abundance (29.14%) in the salivary stone samples, followed by *Capnocytophaga* (9.12%), *Aggregatibacter* (7.7%), and *Neisseria* (7.22%). *Streptococcus* was significantly more abundant in saliva samples from patients compared to those from healthy individuals (*P* = 0.028).

**Table 2:** Top 10 genera by relative abundance in bacterial taxa.

Order	Phylum	Genus	Control Saliva (%)	Patient Saliva (%)	Salivary Stones (%)	Gram staining	Aerobic/anaerobic
1	Firmicutes	*Streptococcus*	27.02	32.22	29.14	+	Facultative anaerobe
2	Actinobacteria	*Rothia*	25.53	30.77	0.86	+	Obligate aerobe
3	Actinobacteria	*Actinomyces*	10.77	8.93	2.73	+	Facultative anaerobe
4	Proteobacteria	*Neisseria*	9.35	6.04	7.22	−	Obligate aerobe
5	Bacteroidetes	*Capnocytophaga*	0.98	0.61	9.12	−	Facultative anaerobe
6	Proteobacteria	*Aggregatibacter*	0.01	0.02	7.7	−	Facultative anaerobe
7	Proteobacteria	*Pseudomonas*	0.03	0.04	6.31	−	Facultative anaerobe
8	Proteobacteria	*Haemophilus*	2.76	4.08	4.91	−	Facultative anaerobe
9	Proteobacteria	*Lautropia*	4.26	2.56	0.09	−	Facultative anaerobe
10	Bacteroidetes	*Porphyromonas*	3.47	1.23	0.52	−	Obligate anaerobe

Relative abundance of bacterial taxa in saliva from healthy individuals, saliva from patients, and salivary stones.

In the saliva samples, Firmicutes was the phylum with the highest number of genera, accounting for 27 (42%) and 33 (45%) of the total number of genera in the control saliva and stone saliva samples, respectively (Fig. 2C). In salivary stone samples, there were 16 genera (32.65%) of Firmicutes and 18 genera (36.73%) of Proteobacteria, accounting for 69.39% of the total number of genera. In the Venn diagram based on genera (Fig. 2D, Supplementary Table S3), saliva samples from patients and from healthy individuals can be observed to share a substantial proportion of genera (95.31% in saliva from healthy individuals, 83.56% in saliva from patients), and salivary stone samples had 29 (59.18%) of 49 genera in common with the saliva samples.

### Differential abundance between groups using ANCOM-BC

**Figure 3:**
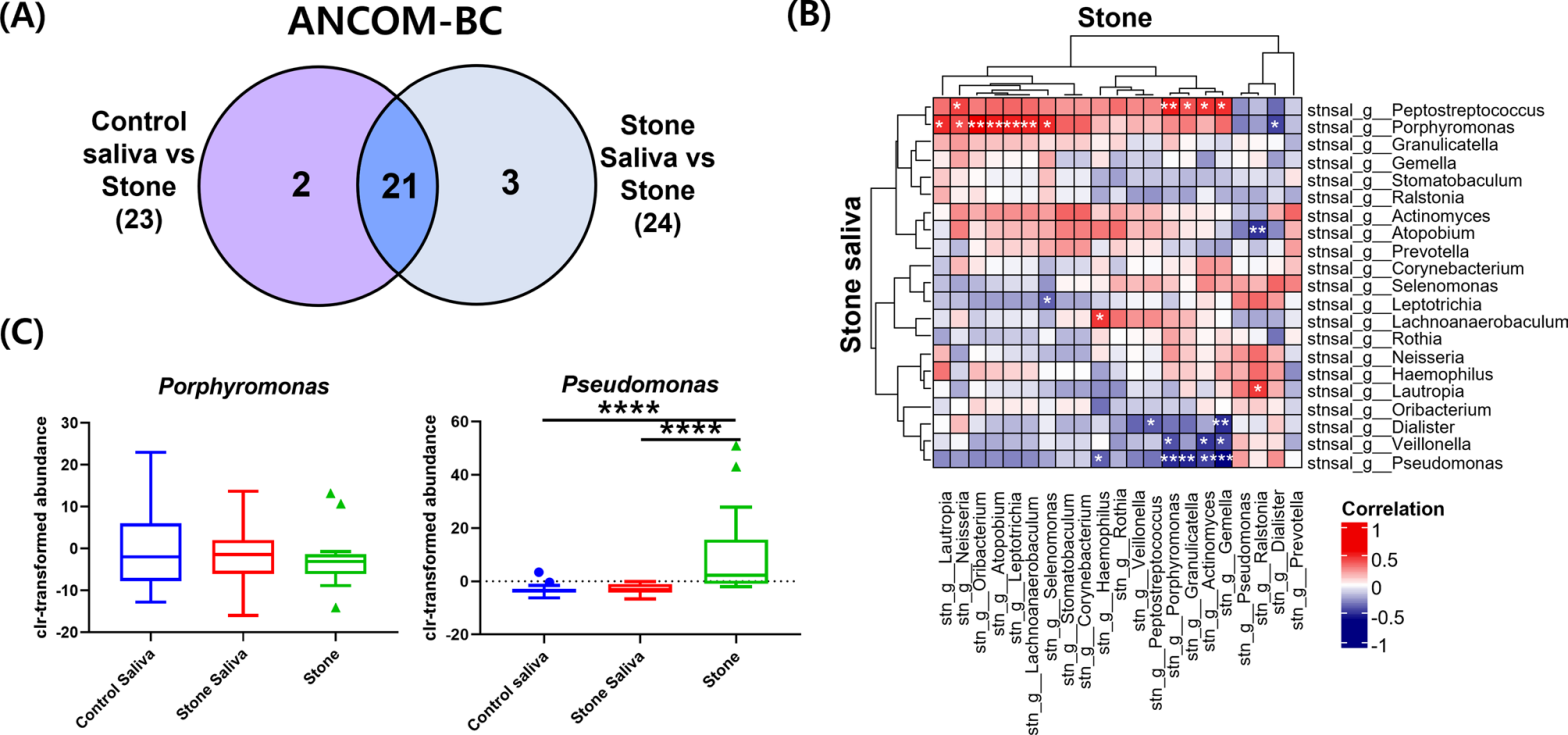
Correlation of the 21 genera between saliva and salivary stones. (**A**) Venn diagram of the genera showing the overall overlap between groups after obtaining 21 genera by Analysis of Compositions of Microbiomes with Bias Correction (ANCOM-BC). Significant differences between saliva from healthy individuals and salivary stones were observed in 23 genera, and between saliva from patients and salivary stones in 24, genera. No genera showed a statistically significant difference in abundance between saliva samples from patients and from healthy individuals. (**B**) Correlation plots between saliva from patients and salivary stones for 21 genera. Positive and negative correlations are represented by blue and red, respectively. Significant correlations are represented by asterisks. (*P < 0.05, **P < 0.01, ***P < 0.001) (**C**) Box plot of *Peptostreptococcus*, *Porphyromonas*, and *Pseudomonas* representing the centered-log ratio-transformed abundance. A Wilcoxon rank-sum test was used to assess differences in alpha-diversity between groups (*P < 0.05, **P < 0.01, ***P < 0.001, ****P < 0.0001).

Differential abundance analysis of salivary microbial communities revealed that participants with and without salivary stones shared similar microbial abundances, as revealed by an analysis of microbiome compositions with bias correction (ANCOM-BC). No genera showed a significant difference in abundance between the control saliva and stone saliva (Fig. 3A, Supplementary Table S4). In contrast, differential abundance analysis between control or stone saliva and stones revealed 23 and 24 distinct genera, respectively, of which 21 were common. These results indicate a significant difference between the microbial communities in saliva and in salivary stone samples.

Twenty-one commonly selected genera (Fig. 3B) were used to compare the microbial environments present in saliva and salivary stone samples. Several genera found in saliva samples, such as *Peptostreptococcus*, *Porphyromonas*, and *Pseudomonas* in stone saliva had a significant correlation with those found in the salivary stone samples. The correlation was positive in the case of *Peptostreptococcus* and *Porphyromonas*. In contrast, *Pseudomonas* in saliva samples from patients were negatively correlated with *Porphyromonas*, *Granulicatella*, *Actinomyces*, and *Gemella* in salivary stone samples. *Porphyromonas* showed a lower abundance in salivary stone samples compared to saliva samples, while *Pseudomonas* showed a significantly higher abundance compared to that in saliva samples from patients (Fig. 3C). There was no significant trend in the correlation between saliva samples from patients and from healthy individuals, whereas there was a strong positive correlation within the salivary stone samples among all genera, except *Dialister*, *Pseudomonas*, and *Ralstonia* (Supplementary Fig. S1).

### PICRUSt predicted KEGG function pathways

**Figure 4:**
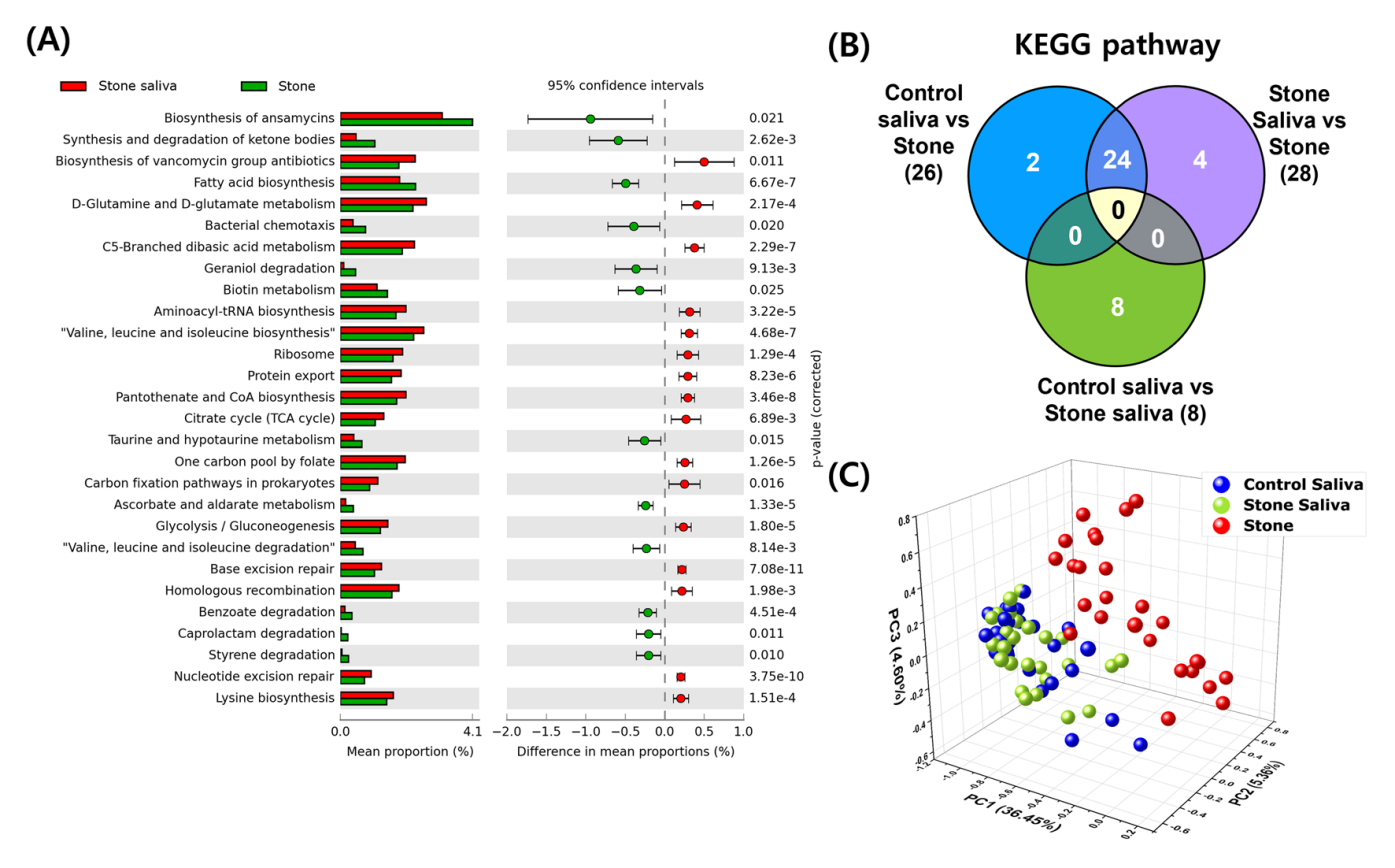
PICRUSt predicted KEGG function between the groups. Error bar plots for two-group analysis comparisons of PICRUSt-predicted KEGG function data using Welch's t-test for the two groups based on (**A**) saliva from patients and salivary stones. A bar graph with expanded error bars was used to compare the two groups, and only predicted functions with *P* < 0.05 are displayed. The bar plots on the left show the average proportion of each KEGG pathway, whereas the dotted plots on the right show the differences in the average proportions between the two groups. (**B**) Venn diagram showing the overall overlap of the KEGG pathways identified using PICRUSt. Significant differences were observed in 8, 26, and 28 KEGG pathways, respectively, between saliva from healthy individuals and patients, saliva from healthy individuals and salivary stones, and saliva from patients and salivary stones. (**C**) Principal component analysis (PCA) of the predicted functional features based on the KEGG pathways using PICRUSt. Permission has been obtained from Kanehisa laboratories for using KEGG pathway database^15^.

PICRUSt analysis revealed eight Kyoto Encyclopedia of Genes and Genomes (KEGG) pathways with significant differences between saliva samples from patients and from healthy individuals, and 24 common significantly different KEGG pathways: 26 pathways for saliva from healthy individuals versus salivary stones, and 28 pathways for saliva from patients versus salivary stones (Fig. 4A,B; Supplementary Fig. S2). The eight pathways showing significant differences between saliva samples from patients and from healthy individuals were different from those showing differences between the saliva and salivary stone samples (Fig. 4B, Supplementary Fig. S2). In addition, PCA of the KEGG pathways from PICRUSt revealed no difference between the saliva samples from patients and from healthy individuals, but saliva and salivary stone samples could be distinguished from one another (Fig. 4C). Therefore, it can be concluded that the oral environment in which salivary stones form is not different from that of the saliva from healthy individuals, whereas the environment within the stone differs substantially from that in the saliva.

### Correlation with genus and KEGG pathway

**Figure 5:**
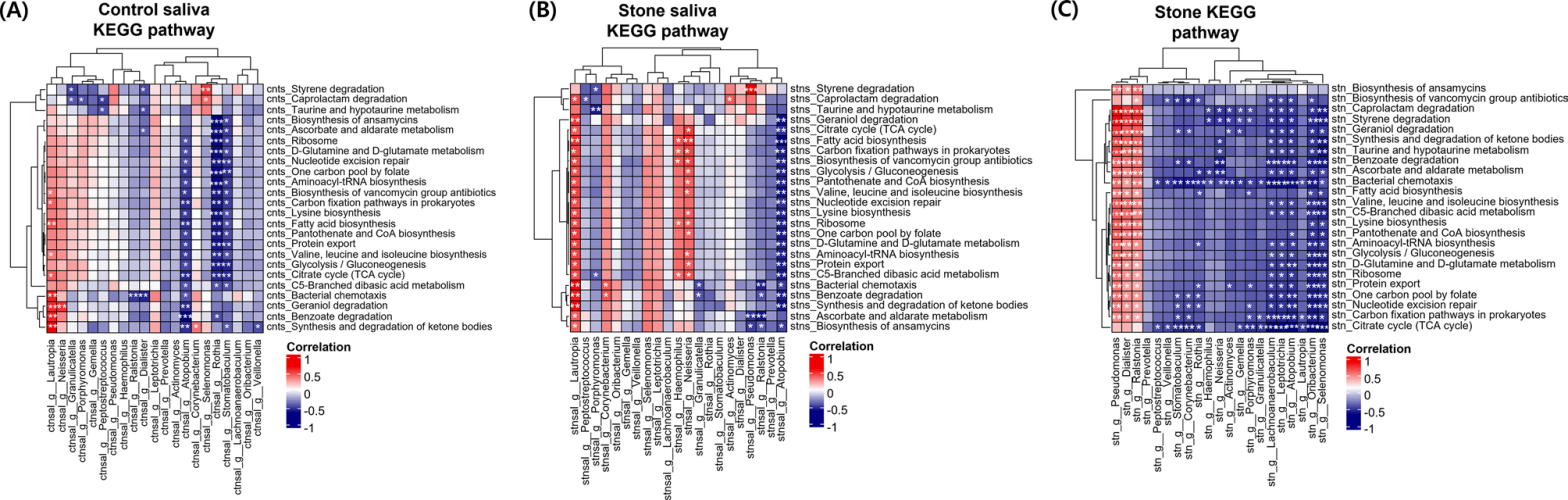
Correlation between the 21 genera and the 24 KEGG pathways. Correlation plots between 21 genera and 24 KEGG pathways for (**A**) control saliva from healthy individuals, (**B**) saliva from patients, and (**C**) salivary stones. Positive and negative correlations are shown in blue and red, respectively. Significant correlations are indicated by asterisks. (*P < 0.05, **P < 0.01, ***P < 0.001).

In the correlation plot between 21 genera and 24 KEGG pathways for saliva samples from patients and from healthy individuals (Fig. 5A,B), *Lautropia* and *Neisseria* positively correlated with pathways that showed significant differences in the PICRUSt analysis between saliva and salivary stone samples, whereas *Atopobium* showed a significant negative correlation between genera and pathways. In the correlation plot for salivary stone samples, the 24 pathways showed a significant positive correlation with *Pseudomonas*, *Dialister*, and *Ralstonia*, and a strong negative correlation with other genera (Fig. 5C). In particular, the bacterial chemotaxis pathway strongly correlated with 21 genera (marked with asterisks).

## Discussion

To the best of our knowledge, this is the first study to compare the core microbiomes of saliva from healthy adults and patients with sialolithiasis, and from salivary stones using 16S rRNA gene sequencing. To establish an association between oral and salivary stone microbial community profiles, we determined the relative abundances of microbial communities in the saliva of healthy adults and in the saliva and salivary stones from adults with sialolithiasis. We found three novel microbiological features of sialolithiasis. First, in the comparison of bacterial diversity or abundance between saliva and salivary stones, the stones showed a markedly decreased diversity. Second, ANCOM-BC was used to identify genera with differential abundance in the saliva of patients and in salivary stones, and the correlation between them was determined. *Peptostreptococcus* and *Porphyromonas* abundance in the saliva from patients positively correlated with that observed in salivary stones. In contrast, the abundance of *Pseudomonas* in saliva from patients was negatively correlated with that observed in salivary stones. Lastly, we reported KEGG pathways associated with salivary stones and found that bacterial chemotaxis and the citrate cycle (TCA cycle) had a significant negative correlation in most of the genera present in salivary stones, but not in those in saliva. Microbiome analysis of saliva from adults with and without salivary stones revealed no differences. Nevertheless, our data allowed us to identify differences in the microbial environments of the saliva and of salivary stones.

Several studies have suggested that salivary stones are associated with the presence of bacteria^11,16–18^. Comparison of chronic obstructive sialadenitis of the submandibular gland and the healthy part of the submandibular gland with fluorescence in situ hybridization and confocal laser scanning microscopy revealed morphological evidence of a bacterial biofilm^16^. Oral *Streptococcus* species were identified in the salivary stones associated with chronic obstructive sialadenitis of the submandibular gland^11^. In another study, 15 salivary stones from 12 patients were examined for the presence of bacteria using optical and scanning electron microscopy, and qPCR revealed the presence of bacterial DNA^12^. These findings support the “retrograde theory” proposed by Marchal et al. postulating that food, bacteria, and foreign objects migrate from the oral cavity to the salivary duct, and that biofilms could be responsible for stone formation^18^.

An essential feature of the microbial environment of salivary stones is its low diversity. This hypothesis was also supported by a comparison of the alpha diversity indices. The richness found in salivary stones was lower than that found in the saliva. Additionally, the Pielou evenness index^19^, which provides a measure of community species evenness, had a value of 0.58 for saliva samples from healthy individuals, 0.56 for saliva samples from patients, and 0.37 (the lowest evenness) for salivary stones. This indicates that, although numerous species are present in saliva, fewer species occur in salivary stones compared to the saliva, and they are unevenly distributed. This is considered to be because saliva is continuously being produced and flowing, and therefore being exposed to various microbes from the outside environment, but the stones form a biofilm within the salivary duct that converts them into closed systems with decreased richness and evenness.

*Streptococcus*, *Capnocytophage*, and *Aggregatibacter* are typically facultatively anaerobic and proliferate easily in the salivary duct environment, where the oxygen supply is insufficient. On the other hand, salivary stones resulting from biofilm formation are unfavorable for the growth of aerobic microbes due to oxygen limitations. Bacteria growing in biofilms are less susceptible to growth-dependent antibiotics because the depletion of nutrients and oxygen causes some bacteria to enter a stationary state^20^. Infections associated with microbial biofilms have been shown to involve hypoxic or anoxic conditions in the vicinity of biofilms^21^. Based on the “retrograde theory,” stones represent a hypoxic/anoxic environment with a microbial community living surrounded by an EPS matrix, and are negatively correlated with *Pseudomonas*^18^. In this study, *Streptococcus* was the most prevalent genus in the salivary stones. However, analysis of the microbial environment of saliva showed that *Streptococcus* is a prevalent genus not only in salivary stones but in saliva as well. In agreement with our results, a previous study comparing the oral microbiome in tonsils and in saliva showed that *Streptococcus* was the most dominant genus in saliva^22^.

*Porphyromonas*, *Granulicatella*, *Actinomyces*, and *Gemella* in salivary stones had a strong positive correlation with *Peptostreptococcus* and a strong negative correlation with *Pseudomonas* in saliva from patients. *Porphyromonas*, *Granulicatella*, *Actinomyces*, and *Gemella* are commensal anaerobic organisms found in the oral cavity in humans. *Peptostreptococcus*, which has a strong positive correlation with these bacteria, is also anaerobic and found in the oral cavity^23^. In contrast, *Pseudomonas* are facultatively anaerobic and have a strong negative correlation with the microbes found in salivary stones. *Porphyromonas* in saliva from patients was positively correlated with *Lautropia*, *Neisseria*, *Oribacterium*, *Atopobium*, *Leptotrichia*, *Lachnoanaerobaculum*, and *Selenomonas* in salivary stones. *Porphyromonas* is an anaerobic Gram-negative bacterium found in the oral cavity, respiratory tract, and gastrointestinal tract, particularly in the salivary microbiome. *Porphyromonas gingivalis*, which forms a multispecies dental calculus biofilm in the oral cavity, has an extracellular structure that stably adheres to the oral cavity, locally invades periodontal tissue, and destroys tooth tissue by evading the defense mechanisms^24^. These bacterial characteristics may positively correlate with the environment of salivary stones. As shown in previous studies^22^, *Streptococcus* is the most abundant bacterium in salivary stones, and is also commonly found in the saliva. Some bacteria exhibited a significant correlation with saliva, demonstrating the influence of saliva on salivary stones.

Thus, the microbiomes of salivary stones and saliva in the salivary duct are quite dissimilar, even though salivary stones share some microbes and are influenced by saliva. Salivary stones receive oxygen and nutrients from the surrounding oral environment, forming their own environment. A salivary stone is an amorphous mineral composed of organic substances such as collagen, glycoproteins, amino acids, and carbohydrates, as well as inorganic components such as hydroxyapatite, carbonate apatite, witlockite, and brushite^25^.

Comparisons between saliva and salivary stones revealed differences in five major functional characteristics: antibiotic biosynthesis and degradation, metabolism and degradation of organic compounds, amino acid metabolism and biosynthesis, cellular metabolism and processes, and cellular regulation and maintenance. The metabolism and degradation of organic compounds and cellular metabolism and processes were observed to be significantly enhanced in salivary stones. These bacterial functions are involved in the degradation of the organic chemicals constituting the stone. The center of the stone is dominated by organic substances, and its periphery by organic and inorganic substances. The decomposition of organic chemicals occurs in the core of the stone.

*Lautropia* and *Atopobium* were the genera in the saliva samples that showed a significant correlation with the 24 KEGG pathways with significant differences between saliva and salivary stones according to PICRUSt. *Lautropia* had a positive correlation with 24 KEGG pathways, whereas *Atopobium* had a negative correlation in saliva. *Lautropia* and *Atopobium* showed a significant positive correlation with *Porphyromonas* in saliva samples from patients. *Lautropia* is a relatively lesser-known genus, and some species have been reported to be found in the oral cavity, especially the gingival margin^26^. *Porphyromonas* and *Lautropia*, both found in the gingiva, are associated with gingival calculi. *Atopobium* is a genus found in the oral cavity, vaginal canal, and gastrointestinal tract, and which contributes to the intestinal metabolic activity, is involved in the metabolism of dietary components, and plays a role in nutrient processing^27,28^.

In the stone KEGG pathway, bacterial chemotaxis and the TCA cycle were negatively correlated with most genera, and this was specific to salivary stones. Bacterial chemotaxis, which is involved in pathogenicity, symbiosis, biofilm formation and stability, and the maintenance of bacteria in their optimal environmental niche, is a movement bias toward environments containing high concentrations of beneficial chemicals or low concentrations of toxic chemicals^29^. The TCA cycle is an important aerobic pathway involved in the final steps of carbohydrate and fatty acid oxidation. The fact that most of the genera found in in salivary stones have a strong negative correlation with these pathways indicates that the stone is a non-growing biofilm due to nutrient and oxygen depletion, and is in a stationary state^20^.

This study had several limitations. First, the sample size was relatively small, and this limitation may hinder the statistical significance of the findings. Additionally, the cross-sectional design of the study only allowed us to evaluate associations with the microbial composition at a specific point in time, not establishing a causal relationship. Future research involving longitudinal studies could provide more information on how microbial communities correlate with salivary stone formation. Interventional studies would be necessary to determine the causal relationships between specific microbial components and stone formation. Second, the analysis in this study targets subsets of the hypervariable regions in 16S rRNA. These subsets are highly homologous or identical between species within the same genus and therefore cannot be used to determine exact species. Consequently, further studies at the species level should be conducted to precisely identify the association between the microbiome and sialolith formation.

In conclusion, we compared the microbial composition of saliva and salivary stones from individuals with and without salivary stones and found that bacterial communities were similar in the saliva of patients and healthy individuals. According to the retrograde theory, a salivary stone is a biofilm that forms in the salivary duct and creates an isolated environment. Although salivary stones are quite different from the saliva in terms of diversity and bacterial composition, some microbes showed associations, suggesting that these microbes interact closely. Our findings shed light on the intricate relationship between the oral microbiome and salivary stone formation. Our preliminary study suggests that it is possible to inhibit or alter the growth of salivary stones in the salivary duct by modulating the microbial community present in the saliva, and suggests the possibility of applying this strategy in the future to develop novel treatments for sialolithiasis.

## Materials and methods

### Sample collection

This prospective study was approved by the institutional review board of Ewha Womans University Mokdong Hospital (IRB No. 2021-08-031). Informed consent was obtained from all participants. All the research protocols were performed in accordance with the Declaration of Helsinki. Patients who underwent intraoral stone removal at the Ewha Womans University Mokdong Hospital between November and August 2022 were enrolled. The control group was recruited from participants in the national health examination who visited the hospital. The patients’ medical histories (hypertension, diabetes mellitus, cancer, and hepatitis) and anthropometric data (height, weight, and BMI) were collected. Oral hygiene habits (tooth brushing, use of dental floss, water intake, alcohol consumption, and tobacco use) were surveyed. Laboratory data were collected. Oral examination and saliva sample collection were conducted on the morning of the day of the operation in the case of patients. All participants were asked to refrain from eating, drinking, or brushing their teeth for at least 4 h before the collection of saliva samples. A 2 × 2 inch sterile gauze was chewed for 1 min to stimulate and collect saliva, and the gauze was collected in sterile 50 mL eppendorf conical tubes. Subsequently, intraoral stone removal was performed. After performing an incision in the oral mucosal, the submandibular gland duct was exposed, a linear incision was made along the duct, and the stone was exposed. To eliminate contamination from oral bacteria, the stone was washed with sterile PBS and immediately extracted from the duct. The stone was then placed in a conical microtube. Collected tubes were stored at − 80 °C until DNA extraction.

### DNA extraction and amplicon sequencing

DNA was extracted from the saliva and the salivary stone samples using the DNeasy PowerSoil Pro kit (QIAGEN, Venlo, Netherlands)^30,31^. The 16S rDNA was amplified using a V3 forward primer (5′-TCG TCG GCA GCG TCA GAT GTG TAT AAG AGA CAG CCT ACG GGN GGC WGC AG-3′) and a V4 reverse primer (5′-GTC TCG TGG GCT CGG AGA TGT GTA TAA GAG ACA GGA CTA CHV GGG TAT CTA ATC C-3′). Index PCR with sequencing adapters attached to the amplified DNA was performed using the Nextera XT Index kit (Illumina, San Diego, CA, USA). Following the manufacturer's instructions, 25 cycles were performed in the amplicon PCR step and eight cycles in the index PCR step. After the quantification, normalization, and pooling of the libraries, each amplicon was sequenced using a MiSeq Reagent Kit (v3, 600 cycles, Illumina). All raw sequences derived from this experiment were submitted to the Short Read Archive of NCBI and can be found under the BioProject accession number #PRJNA948151.

### Analysis of bacterial compositions of microbiomes

In total, 2,286,166 raw reads were generated from the saliva and stone samples, with an average of 28,224 reads (standard deviation: 12,895) per sample. Paired-end 16S rRNA gene sequences were entered into Quantitative Insights into Microbial Ecology (QIIME2 v2021.4)^32^. Adapter sequences were removed using Cutadapt version 3.4.0^33^. The forward and reverse reads were truncated at 200 and 260 bases, respectively, based on a Phred (Q) score of 30. Reads were filtered for quality and chimeric reads using DADA2^34^ with manual parameters (trim-left-f 0, trim-left-r 0, trunc-len-f 260, trunc-len-r 200, trunc-q 2, max-ee-f 3, and max-ee-r 3). The DADA2 algorithm was used for modeling and correcting Illumina-sequenced amplicon errors and was then used for the identification of amplicon sequencing variants (ASVs). Taxonomic classification was performed using naïve Bayes classifiers trained on the extracted V3-V4 region from the SILVA 138 database^35^. If taxa could not be assigned to each level owing to a lack of or redundant sequences in the database, the taxon was assigned to the next higher level, as indicated in parentheses.

### Statistical analysis

Alpha diversity was calculated for a rarified dataset (15,093 saliva and 15, 688 reads tonsillar tissue reads) using the QIIME pipeline. Observed OTUs, Chao1, Shannon, and Simpson indices were used to calculate the species diversity between the groups. Statistical comparison of alpha diversities between groups was performed using the Kruskal**–**Wallis test, and *P* values were adjusted with the Benjamini**–**Hochberg correction. The resultant distance matrix was applied to generate PCA plots based on “Bray**–**Curtis dissimilarity.” To statistically evaluate the significance of grouping, analysis of similarities (ANOSIM) was performed using the R package “vegan” (distance: “Euclidean”, Permutations: 999).

To distinguish whether specific genera were differentially abundant across particular groups of individuals, an analysis of the composition of microbiomes with bias correction (ANCOM-BC) was performed using ANCOMBC 2.0.3^36^. As part of the ANCOM-BC, the Benjamini–Hochberg method was used to correct the *P*-values for multiple testing. A cutoff of *P*_adj_ < 0.05 was used to assess significance. To assess the metabolic potential of the microbial communities found in the three different groups, the OTUs were imported into PICRUSt2^37^ and the Kyoto Encyclopedia of Genes and Genomes (KEGG) database was used to predict the functional gene content of the various microbial communities. The correlation of genera between saliva and salivary stones was further analyzed using Spearman’s correlation coefficient. All statistical analyses were performed using R version 4.2.2. *P*-values < 0.05 were considered statistically significant.

### Supplementary Information

Supplementary Information.

## Data Availability

All data is available and provided as supplementary material and all raw sequences derived from this experiment were submitted to the Short Read Archive of NCBI and can be found under BioProject accession number #PRJNA948151”.
