# Supplementary material for: Microbiomic association between the saliva and salivary stone in patients with sialolithiasis
Source: Sci Rep. 2024 Apr 22;14:9184. doi: 10.1038/s41598-024-59546-x (PMC11035639; doi:10.1038/s41598-024-59546-x)
Supplement: Supplementary file 1 — Supplementary Information. [file 41598_2024_59546_MOESM1_ESM.pptx]

## Slide 1
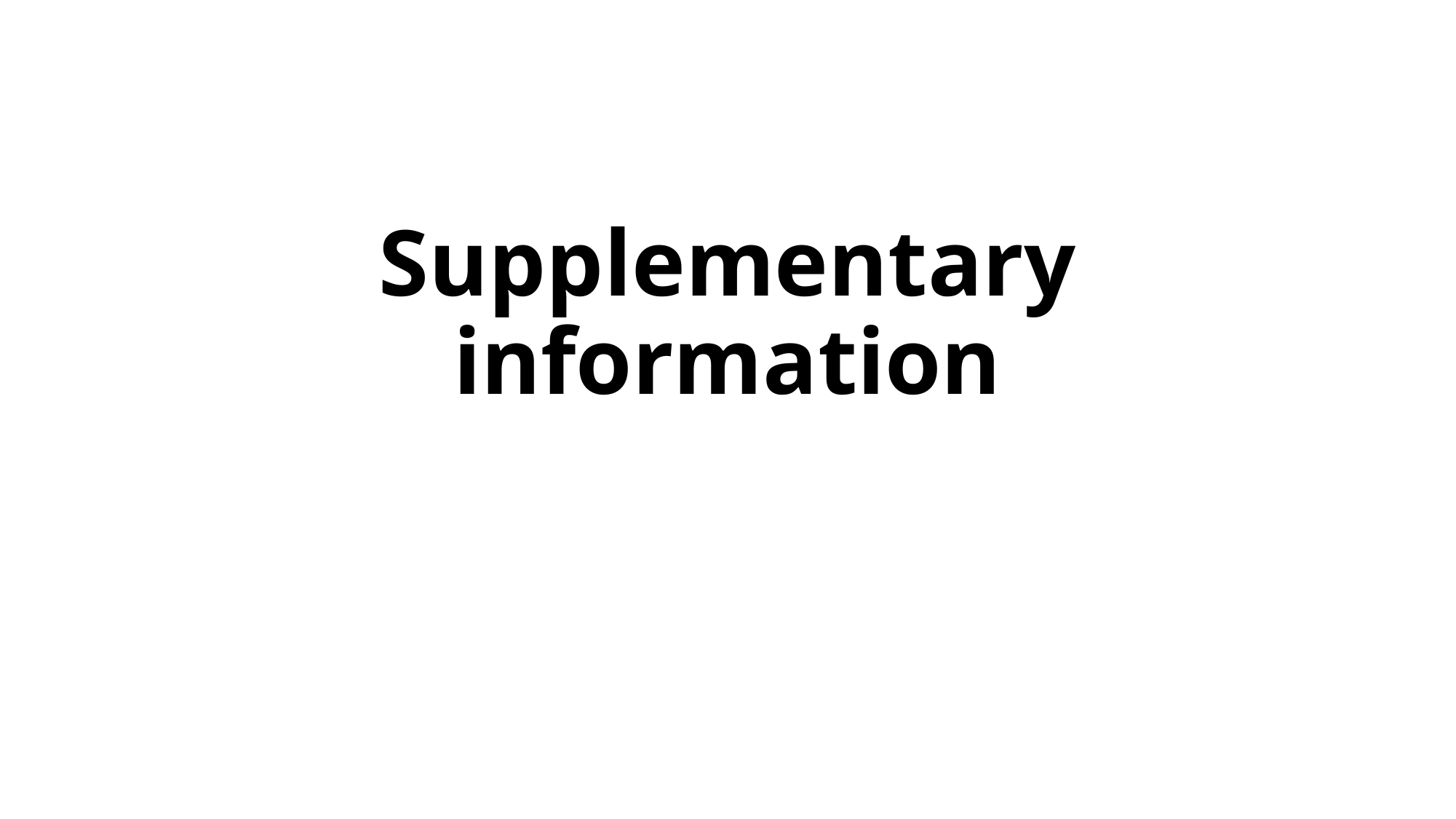

# Supplementary information

## Slide 2
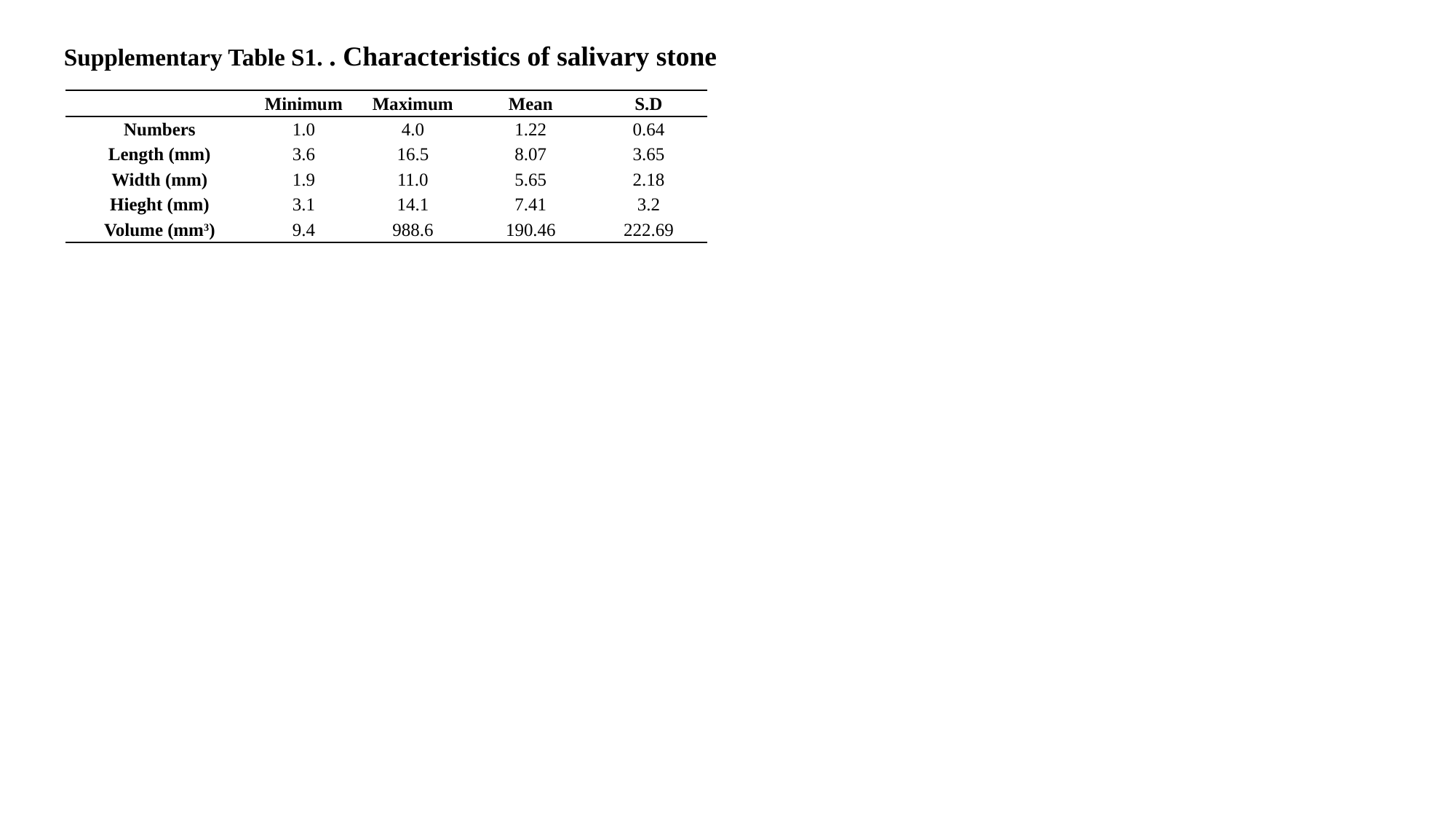

Supplementary Table S1. . Characteristics of salivary stone
| | Minimum | Maximum | Mean | S.D |
| --- | --- | --- | --- | --- |
| Numbers | 1.0 | 4.0 | 1.22 | 0.64 |
| Length (mm) | 3.6 | 16.5 | 8.07 | 3.65 |
| Width (mm) | 1.9 | 11.0 | 5.65 | 2.18 |
| Hieght (mm) | 3.1 | 14.1 | 7.41 | 3.2 |
| Volume (mm3) | 9.4 | 988.6 | 190.46 | 222.69 |

## Slide 3
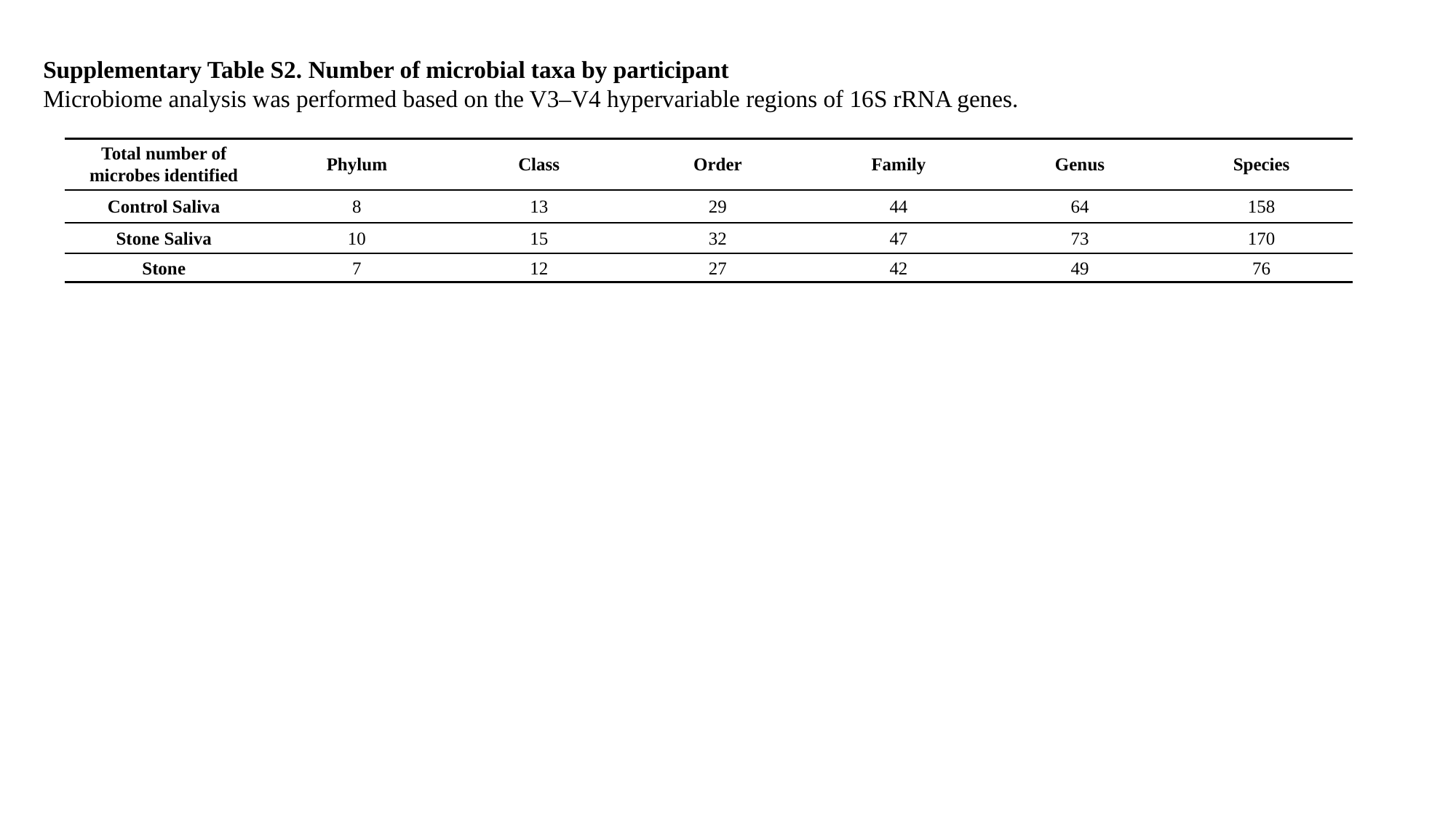

Supplementary Table S2. Number of microbial taxa by participant
Microbiome analysis was performed based on the V3–V4 hypervariable regions of 16S rRNA genes.
| Total number of microbes identified | Phylum | Class | Order | Family | Genus | Species |
| --- | --- | --- | --- | --- | --- | --- |
| Control Saliva | 8 | 13 | 29 | 44 | 64 | 158 |
| Stone Saliva | 10 | 15 | 32 | 47 | 73 | 170 |
| Stone | 7 | 12 | 27 | 42 | 49 | 76 |

## Slide 4
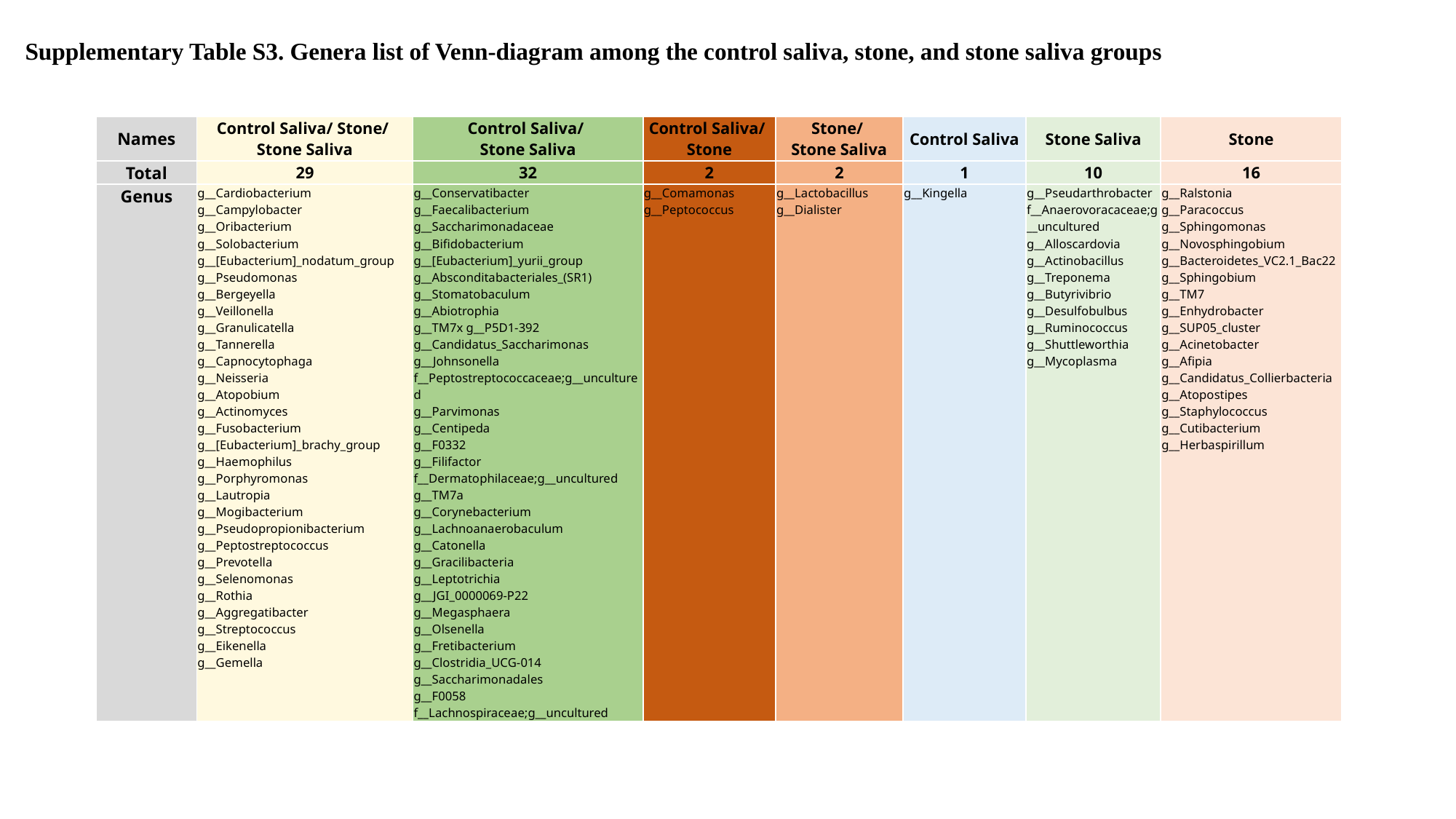

Supplementary Table S3. Genera list of Venn-diagram among the control saliva, stone, and stone saliva groups
| Names | Control Saliva/ Stone/ Stone Saliva | Control Saliva/ Stone Saliva | Control Saliva/ Stone | Stone/ Stone Saliva | Control Saliva | Stone Saliva | Stone |
| --- | --- | --- | --- | --- | --- | --- | --- |
| Total | 29 | 32 | 2 | 2 | 1 | 10 | 16 |
| Genus | g\_\_Cardiobacterium g\_\_Campylobacter g\_\_Oribacterium g\_\_Solobacterium g\_\_[Eubacterium]\_nodatum\_group g\_\_Pseudomonas g\_\_Bergeyella g\_\_Veillonella g\_\_Granulicatella g\_\_Tannerella g\_\_Capnocytophaga g\_\_Neisseria g\_\_Atopobium g\_\_Actinomyces g\_\_Fusobacterium g\_\_[Eubacterium]\_brachy\_group g\_\_Haemophilus g\_\_Porphyromonas g\_\_Lautropia g\_\_Mogibacterium g\_\_Pseudopropionibacterium g\_\_Peptostreptococcus g\_\_Prevotella g\_\_Selenomonas g\_\_Rothia g\_\_Aggregatibacter g\_\_Streptococcus g\_\_Eikenella g\_\_Gemella | g\_\_Conservatibacter g\_\_Faecalibacterium g\_\_Saccharimonadaceae g\_\_Bifidobacterium g\_\_[Eubacterium]\_yurii\_group g\_\_Absconditabacteriales\_(SR1) g\_\_Stomatobaculum g\_\_Abiotrophia g\_\_TM7x g\_\_P5D1-392 g\_\_Candidatus\_Saccharimonas g\_\_Johnsonella f\_\_Peptostreptococcaceae;g\_\_uncultured g\_\_Parvimonas g\_\_Centipeda g\_\_F0332 g\_\_Filifactor f\_\_Dermatophilaceae;g\_\_uncultured g\_\_TM7a g\_\_Corynebacterium g\_\_Lachnoanaerobaculum g\_\_Catonella g\_\_Gracilibacteria g\_\_Leptotrichia g\_\_JGI\_0000069-P22 g\_\_Megasphaera g\_\_Olsenella g\_\_Fretibacterium g\_\_Clostridia\_UCG-014 g\_\_Saccharimonadales g\_\_F0058 f\_\_Lachnospiraceae;g\_\_uncultured | g\_\_Comamonas g\_\_Peptococcus | g\_\_Lactobacillus g\_\_Dialister | g\_\_Kingella | g\_\_Pseudarthrobacter f\_\_Anaerovoracaceae;g\_\_uncultured g\_\_Alloscardovia g\_\_Actinobacillus g\_\_Treponema g\_\_Butyrivibrio g\_\_Desulfobulbus g\_\_Ruminococcus g\_\_Shuttleworthia g\_\_Mycoplasma | g\_\_Ralstonia g\_\_Paracoccus g\_\_Sphingomonas g\_\_Novosphingobium g\_\_Bacteroidetes\_VC2.1\_Bac22 g\_\_Sphingobium g\_\_TM7 g\_\_Enhydrobacter g\_\_SUP05\_cluster g\_\_Acinetobacter g\_\_Afipia g\_\_Candidatus\_Collierbacteria g\_\_Atopostipes g\_\_Staphylococcus g\_\_Cutibacterium g\_\_Herbaspirillum |

## Slide 5
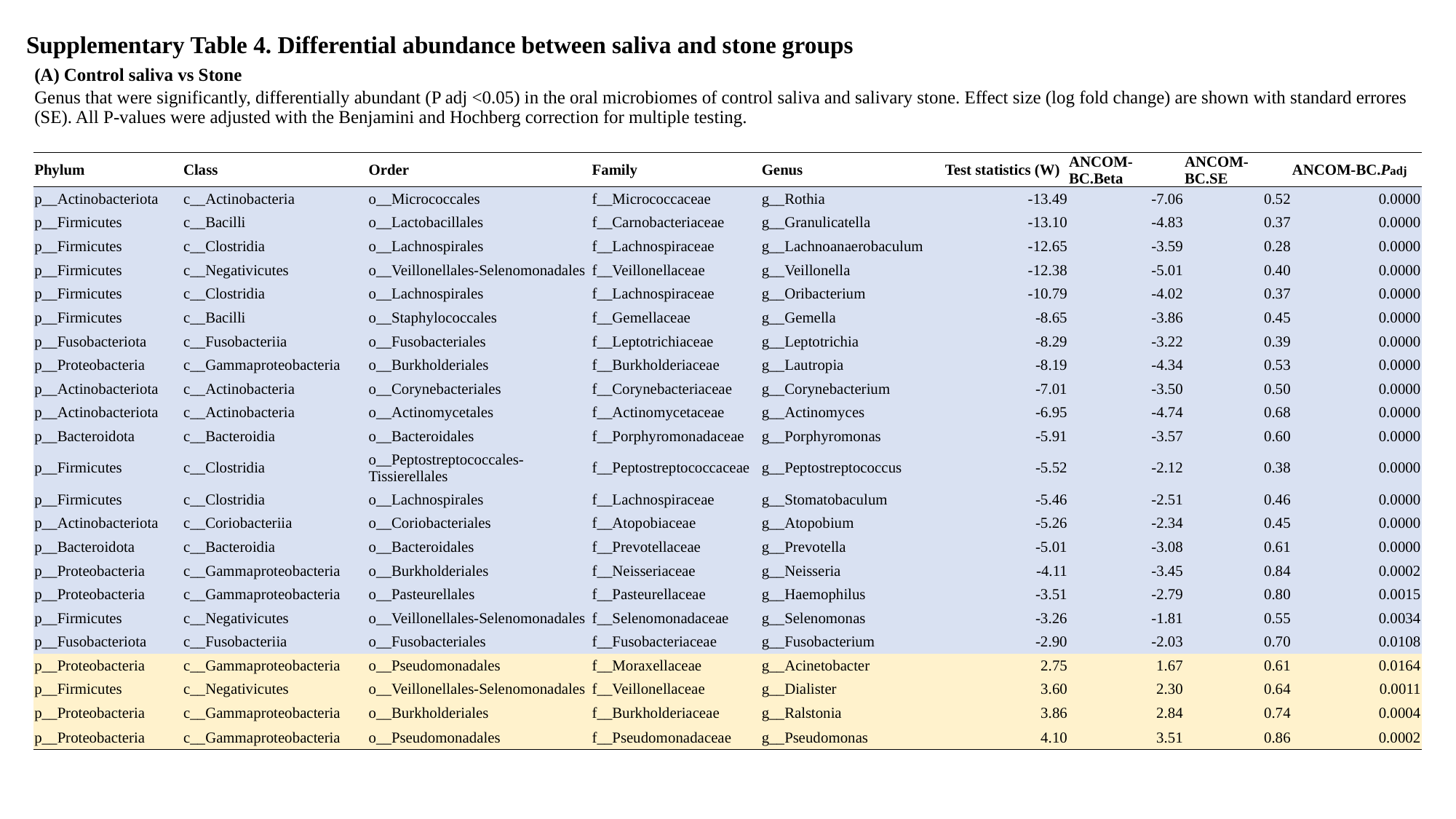

Supplementary Table 4. Differential abundance between saliva and stone groups
| (A) Control saliva vs Stone | | | | | | | | |
| --- | --- | --- | --- | --- | --- | --- | --- | --- |
| Genus that were significantly, differentially abundant (P adj <0.05) in the oral microbiomes of control saliva and salivary stone. Effect size (log fold change) are shown with standard errores (SE). All P-values were adjusted with the Benjamini and Hochberg correction for multiple testing. | | | | | | | | |
| | | | | | | | | |
| Phylum | Class | Order | Family | Genus | Test statistics (W) | ANCOM-BC.Beta | ANCOM-BC.SE | ANCOM-BC.Padj |
| p\_\_Actinobacteriota | c\_\_Actinobacteria | o\_\_Micrococcales | f\_\_Micrococcaceae | g\_\_Rothia | -13.49 | -7.06 | 0.52 | 0.0000 |
| p\_\_Firmicutes | c\_\_Bacilli | o\_\_Lactobacillales | f\_\_Carnobacteriaceae | g\_\_Granulicatella | -13.10 | -4.83 | 0.37 | 0.0000 |
| p\_\_Firmicutes | c\_\_Clostridia | o\_\_Lachnospirales | f\_\_Lachnospiraceae | g\_\_Lachnoanaerobaculum | -12.65 | -3.59 | 0.28 | 0.0000 |
| p\_\_Firmicutes | c\_\_Negativicutes | o\_\_Veillonellales-Selenomonadales | f\_\_Veillonellaceae | g\_\_Veillonella | -12.38 | -5.01 | 0.40 | 0.0000 |
| p\_\_Firmicutes | c\_\_Clostridia | o\_\_Lachnospirales | f\_\_Lachnospiraceae | g\_\_Oribacterium | -10.79 | -4.02 | 0.37 | 0.0000 |
| p\_\_Firmicutes | c\_\_Bacilli | o\_\_Staphylococcales | f\_\_Gemellaceae | g\_\_Gemella | -8.65 | -3.86 | 0.45 | 0.0000 |
| p\_\_Fusobacteriota | c\_\_Fusobacteriia | o\_\_Fusobacteriales | f\_\_Leptotrichiaceae | g\_\_Leptotrichia | -8.29 | -3.22 | 0.39 | 0.0000 |
| p\_\_Proteobacteria | c\_\_Gammaproteobacteria | o\_\_Burkholderiales | f\_\_Burkholderiaceae | g\_\_Lautropia | -8.19 | -4.34 | 0.53 | 0.0000 |
| p\_\_Actinobacteriota | c\_\_Actinobacteria | o\_\_Corynebacteriales | f\_\_Corynebacteriaceae | g\_\_Corynebacterium | -7.01 | -3.50 | 0.50 | 0.0000 |
| p\_\_Actinobacteriota | c\_\_Actinobacteria | o\_\_Actinomycetales | f\_\_Actinomycetaceae | g\_\_Actinomyces | -6.95 | -4.74 | 0.68 | 0.0000 |
| p\_\_Bacteroidota | c\_\_Bacteroidia | o\_\_Bacteroidales | f\_\_Porphyromonadaceae | g\_\_Porphyromonas | -5.91 | -3.57 | 0.60 | 0.0000 |
| p\_\_Firmicutes | c\_\_Clostridia | o\_\_Peptostreptococcales-Tissierellales | f\_\_Peptostreptococcaceae | g\_\_Peptostreptococcus | -5.52 | -2.12 | 0.38 | 0.0000 |
| p\_\_Firmicutes | c\_\_Clostridia | o\_\_Lachnospirales | f\_\_Lachnospiraceae | g\_\_Stomatobaculum | -5.46 | -2.51 | 0.46 | 0.0000 |
| p\_\_Actinobacteriota | c\_\_Coriobacteriia | o\_\_Coriobacteriales | f\_\_Atopobiaceae | g\_\_Atopobium | -5.26 | -2.34 | 0.45 | 0.0000 |
| p\_\_Bacteroidota | c\_\_Bacteroidia | o\_\_Bacteroidales | f\_\_Prevotellaceae | g\_\_Prevotella | -5.01 | -3.08 | 0.61 | 0.0000 |
| p\_\_Proteobacteria | c\_\_Gammaproteobacteria | o\_\_Burkholderiales | f\_\_Neisseriaceae | g\_\_Neisseria | -4.11 | -3.45 | 0.84 | 0.0002 |
| p\_\_Proteobacteria | c\_\_Gammaproteobacteria | o\_\_Pasteurellales | f\_\_Pasteurellaceae | g\_\_Haemophilus | -3.51 | -2.79 | 0.80 | 0.0015 |
| p\_\_Firmicutes | c\_\_Negativicutes | o\_\_Veillonellales-Selenomonadales | f\_\_Selenomonadaceae | g\_\_Selenomonas | -3.26 | -1.81 | 0.55 | 0.0034 |
| p\_\_Fusobacteriota | c\_\_Fusobacteriia | o\_\_Fusobacteriales | f\_\_Fusobacteriaceae | g\_\_Fusobacterium | -2.90 | -2.03 | 0.70 | 0.0108 |
| p\_\_Proteobacteria | c\_\_Gammaproteobacteria | o\_\_Pseudomonadales | f\_\_Moraxellaceae | g\_\_Acinetobacter | 2.75 | 1.67 | 0.61 | 0.0164 |
| p\_\_Firmicutes | c\_\_Negativicutes | o\_\_Veillonellales-Selenomonadales | f\_\_Veillonellaceae | g\_\_Dialister | 3.60 | 2.30 | 0.64 | 0.0011 |
| p\_\_Proteobacteria | c\_\_Gammaproteobacteria | o\_\_Burkholderiales | f\_\_Burkholderiaceae | g\_\_Ralstonia | 3.86 | 2.84 | 0.74 | 0.0004 |
| p\_\_Proteobacteria | c\_\_Gammaproteobacteria | o\_\_Pseudomonadales | f\_\_Pseudomonadaceae | g\_\_Pseudomonas | 4.10 | 3.51 | 0.86 | 0.0002 |

## Slide 6
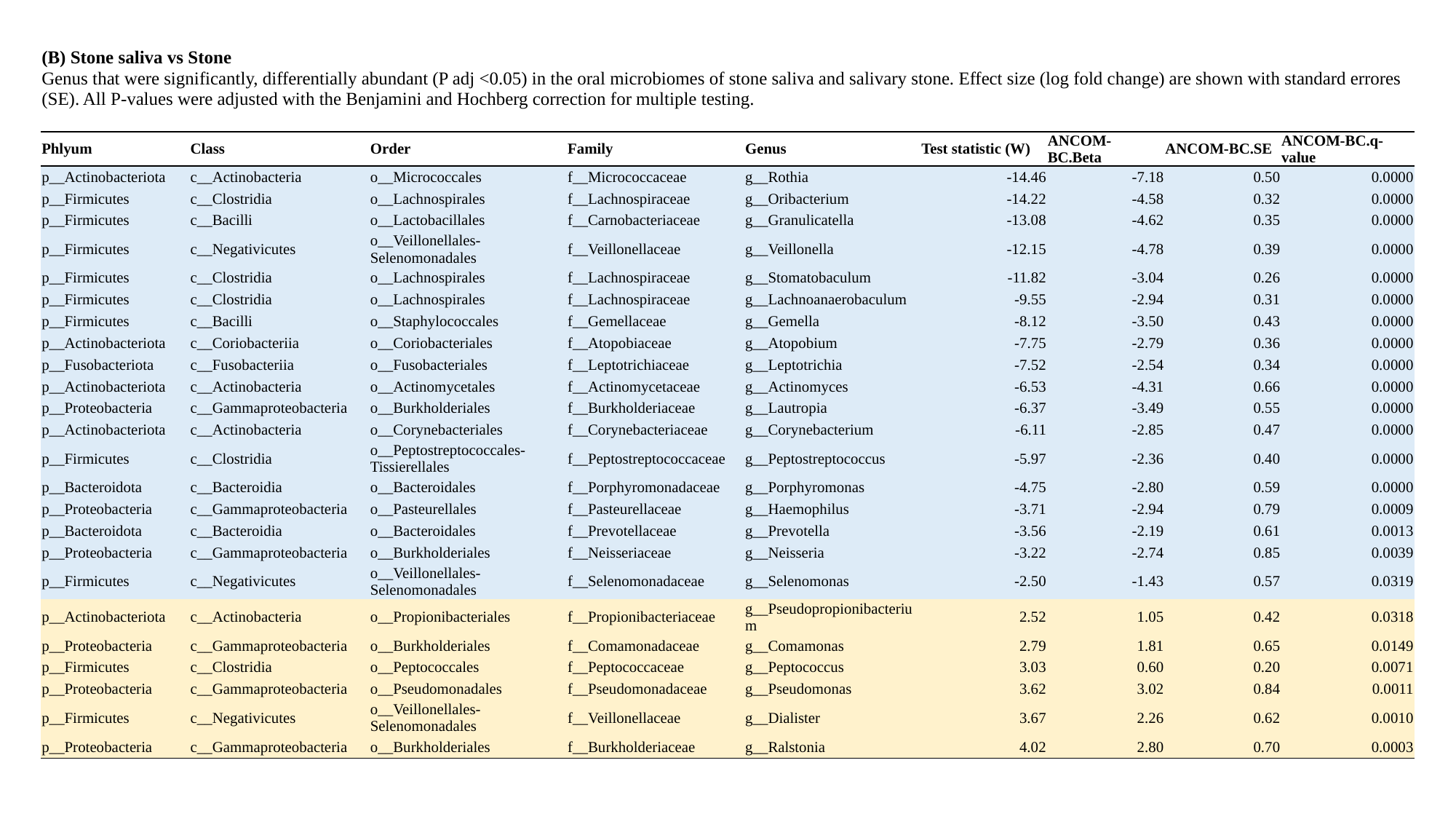

| (B) Stone saliva vs Stone | | | | | | | | |
| --- | --- | --- | --- | --- | --- | --- | --- | --- |
| Genus that were significantly, differentially abundant (P adj <0.05) in the oral microbiomes of stone saliva and salivary stone. Effect size (log fold change) are shown with standard errores (SE). All P-values were adjusted with the Benjamini and Hochberg correction for multiple testing. | | | | | | | | |
| | | | | | | | | |
| Phlyum | Class | Order | Family | Genus | Test statistic (W) | ANCOM-BC.Beta | ANCOM-BC.SE | ANCOM-BC.q-value |
| p\_\_Actinobacteriota | c\_\_Actinobacteria | o\_\_Micrococcales | f\_\_Micrococcaceae | g\_\_Rothia | -14.46 | -7.18 | 0.50 | 0.0000 |
| p\_\_Firmicutes | c\_\_Clostridia | o\_\_Lachnospirales | f\_\_Lachnospiraceae | g\_\_Oribacterium | -14.22 | -4.58 | 0.32 | 0.0000 |
| p\_\_Firmicutes | c\_\_Bacilli | o\_\_Lactobacillales | f\_\_Carnobacteriaceae | g\_\_Granulicatella | -13.08 | -4.62 | 0.35 | 0.0000 |
| p\_\_Firmicutes | c\_\_Negativicutes | o\_\_Veillonellales-Selenomonadales | f\_\_Veillonellaceae | g\_\_Veillonella | -12.15 | -4.78 | 0.39 | 0.0000 |
| p\_\_Firmicutes | c\_\_Clostridia | o\_\_Lachnospirales | f\_\_Lachnospiraceae | g\_\_Stomatobaculum | -11.82 | -3.04 | 0.26 | 0.0000 |
| p\_\_Firmicutes | c\_\_Clostridia | o\_\_Lachnospirales | f\_\_Lachnospiraceae | g\_\_Lachnoanaerobaculum | -9.55 | -2.94 | 0.31 | 0.0000 |
| p\_\_Firmicutes | c\_\_Bacilli | o\_\_Staphylococcales | f\_\_Gemellaceae | g\_\_Gemella | -8.12 | -3.50 | 0.43 | 0.0000 |
| p\_\_Actinobacteriota | c\_\_Coriobacteriia | o\_\_Coriobacteriales | f\_\_Atopobiaceae | g\_\_Atopobium | -7.75 | -2.79 | 0.36 | 0.0000 |
| p\_\_Fusobacteriota | c\_\_Fusobacteriia | o\_\_Fusobacteriales | f\_\_Leptotrichiaceae | g\_\_Leptotrichia | -7.52 | -2.54 | 0.34 | 0.0000 |
| p\_\_Actinobacteriota | c\_\_Actinobacteria | o\_\_Actinomycetales | f\_\_Actinomycetaceae | g\_\_Actinomyces | -6.53 | -4.31 | 0.66 | 0.0000 |
| p\_\_Proteobacteria | c\_\_Gammaproteobacteria | o\_\_Burkholderiales | f\_\_Burkholderiaceae | g\_\_Lautropia | -6.37 | -3.49 | 0.55 | 0.0000 |
| p\_\_Actinobacteriota | c\_\_Actinobacteria | o\_\_Corynebacteriales | f\_\_Corynebacteriaceae | g\_\_Corynebacterium | -6.11 | -2.85 | 0.47 | 0.0000 |
| p\_\_Firmicutes | c\_\_Clostridia | o\_\_Peptostreptococcales-Tissierellales | f\_\_Peptostreptococcaceae | g\_\_Peptostreptococcus | -5.97 | -2.36 | 0.40 | 0.0000 |
| p\_\_Bacteroidota | c\_\_Bacteroidia | o\_\_Bacteroidales | f\_\_Porphyromonadaceae | g\_\_Porphyromonas | -4.75 | -2.80 | 0.59 | 0.0000 |
| p\_\_Proteobacteria | c\_\_Gammaproteobacteria | o\_\_Pasteurellales | f\_\_Pasteurellaceae | g\_\_Haemophilus | -3.71 | -2.94 | 0.79 | 0.0009 |
| p\_\_Bacteroidota | c\_\_Bacteroidia | o\_\_Bacteroidales | f\_\_Prevotellaceae | g\_\_Prevotella | -3.56 | -2.19 | 0.61 | 0.0013 |
| p\_\_Proteobacteria | c\_\_Gammaproteobacteria | o\_\_Burkholderiales | f\_\_Neisseriaceae | g\_\_Neisseria | -3.22 | -2.74 | 0.85 | 0.0039 |
| p\_\_Firmicutes | c\_\_Negativicutes | o\_\_Veillonellales-Selenomonadales | f\_\_Selenomonadaceae | g\_\_Selenomonas | -2.50 | -1.43 | 0.57 | 0.0319 |
| p\_\_Actinobacteriota | c\_\_Actinobacteria | o\_\_Propionibacteriales | f\_\_Propionibacteriaceae | g\_\_Pseudopropionibacterium | 2.52 | 1.05 | 0.42 | 0.0318 |
| p\_\_Proteobacteria | c\_\_Gammaproteobacteria | o\_\_Burkholderiales | f\_\_Comamonadaceae | g\_\_Comamonas | 2.79 | 1.81 | 0.65 | 0.0149 |
| p\_\_Firmicutes | c\_\_Clostridia | o\_\_Peptococcales | f\_\_Peptococcaceae | g\_\_Peptococcus | 3.03 | 0.60 | 0.20 | 0.0071 |
| p\_\_Proteobacteria | c\_\_Gammaproteobacteria | o\_\_Pseudomonadales | f\_\_Pseudomonadaceae | g\_\_Pseudomonas | 3.62 | 3.02 | 0.84 | 0.0011 |
| p\_\_Firmicutes | c\_\_Negativicutes | o\_\_Veillonellales-Selenomonadales | f\_\_Veillonellaceae | g\_\_Dialister | 3.67 | 2.26 | 0.62 | 0.0010 |
| p\_\_Proteobacteria | c\_\_Gammaproteobacteria | o\_\_Burkholderiales | f\_\_Burkholderiaceae | g\_\_Ralstonia | 4.02 | 2.80 | 0.70 | 0.0003 |

## Slide 7
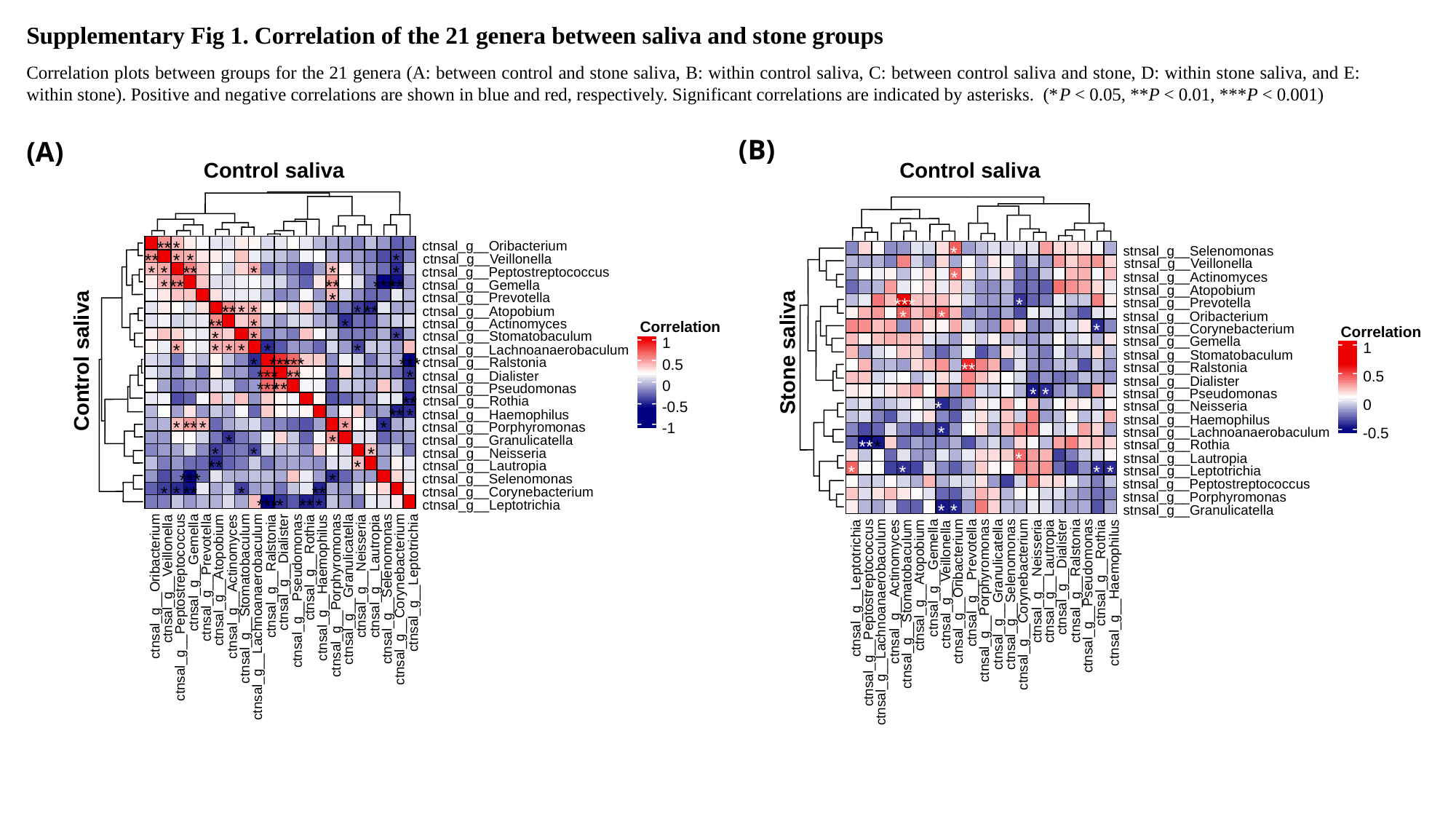

Supplementary Fig 1. Correlation of the 21 genera between saliva and stone groups
Correlation plots between groups for the 21 genera (A: between control and stone saliva, B: within control saliva, C: between control saliva and stone, D: within stone saliva, and E: within stone). Positive and negative correlations are shown in blue and red, respectively. Significant correlations are indicated by asterisks. (*P < 0.05, **P < 0.01, ***P < 0.001)
(B)
(A)
Control saliva
ctnsal_g__Oribacterium
ctnsal_g__Veillonella
ctnsal_g__Peptostreptococcus
ctnsal_g__Gemella
ctnsal_g__Prevotella
ctnsal_g__Atopobium
ctnsal_g__Actinomyces
Correlation
ctnsal_g__Stomatobaculum
1
ctnsal_g__Lachnoanaerobaculum
ctnsal_g__Ralstonia
0.5
ctnsal_g__Dialister
0
ctnsal_g__Pseudomonas
ctnsal_g__Rothia
-0.5
ctnsal_g__Haemophilus
ctnsal_g__Porphyromonas
-1
ctnsal_g__Granulicatella
ctnsal_g__Neisseria
ctnsal_g__Lautropia
ctnsal_g__Selenomonas
ctnsal_g__Corynebacterium
ctnsal_g__Leptotrichia
ctnsal_g__Rothia
ctnsal_g__Dialister
ctnsal_g__Gemella
ctnsal_g__Ralstonia
ctnsal_g__Neisseria
ctnsal_g__Lautropia
ctnsal_g__Prevotella
ctnsal_g__Veillonella
ctnsal_g__Atopobium
ctnsal_g__Leptotrichia
ctnsal_g__Oribacterium
ctnsal_g__Actinomyces
ctnsal_g__Haemophilus
ctnsal_g__Granulicatella
ctnsal_g__Selenomonas
ctnsal_g__Pseudomonas
ctnsal_g__Porphyromonas
ctnsal_g__Stomatobaculum
ctnsal_g__Corynebacterium
ctnsal_g__Peptostreptococcus
ctnsal_g__Lachnoanaerobaculum
**
*
**
*
*
*
*
*
**
*
*
*
*
**
**
***
**
*
**
*
*
*
**
**
*
*
*
*
*
*
*
*
*
*
*
*
***
***
***
***
**
*
***
**
**
**
*
*
**
*
*
*
*
*
*
*
*
**
*
***
*
*
*
**
*
**
***
*
**
*
Control saliva
Control saliva
*
*
***
*
*
*
*
**
*
*
*
*
**
*
*
*
*
*
*
*
*
stnsal_g__Selenomonas
stnsal_g__Veillonella
stnsal_g__Actinomyces
stnsal_g__Atopobium
stnsal_g__Prevotella
stnsal_g__Oribacterium
stnsal_g__Corynebacterium
Correlation
stnsal_g__Gemella
1
stnsal_g__Stomatobaculum
stnsal_g__Ralstonia
0.5
stnsal_g__Dialister
stnsal_g__Pseudomonas
0
stnsal_g__Neisseria
stnsal_g__Haemophilus
stnsal_g__Lachnoanaerobaculum
-0.5
stnsal_g__Rothia
stnsal_g__Lautropia
stnsal_g__Leptotrichia
stnsal_g__Peptostreptococcus
stnsal_g__Porphyromonas
stnsal_g__Granulicatella
ctnsal_g__Rothia
ctnsal_g__Dialister
ctnsal_g__Gemella
ctnsal_g__Neisseria
ctnsal_g__Lautropia
ctnsal_g__Ralstonia
ctnsal_g__Prevotella
ctnsal_g__Veillonella
ctnsal_g__Atopobium
ctnsal_g__Leptotrichia
ctnsal_g__Actinomyces
ctnsal_g__Oribacterium
ctnsal_g__Haemophilus
ctnsal_g__Granulicatella
ctnsal_g__Selenomonas
ctnsal_g__Pseudomonas
ctnsal_g__Porphyromonas
ctnsal_g__Stomatobaculum
ctnsal_g__Corynebacterium
ctnsal_g__Peptostreptococcus
ctnsal_g__Lachnoanaerobaculum
Stone saliva
Stone saliva

## Slide 8
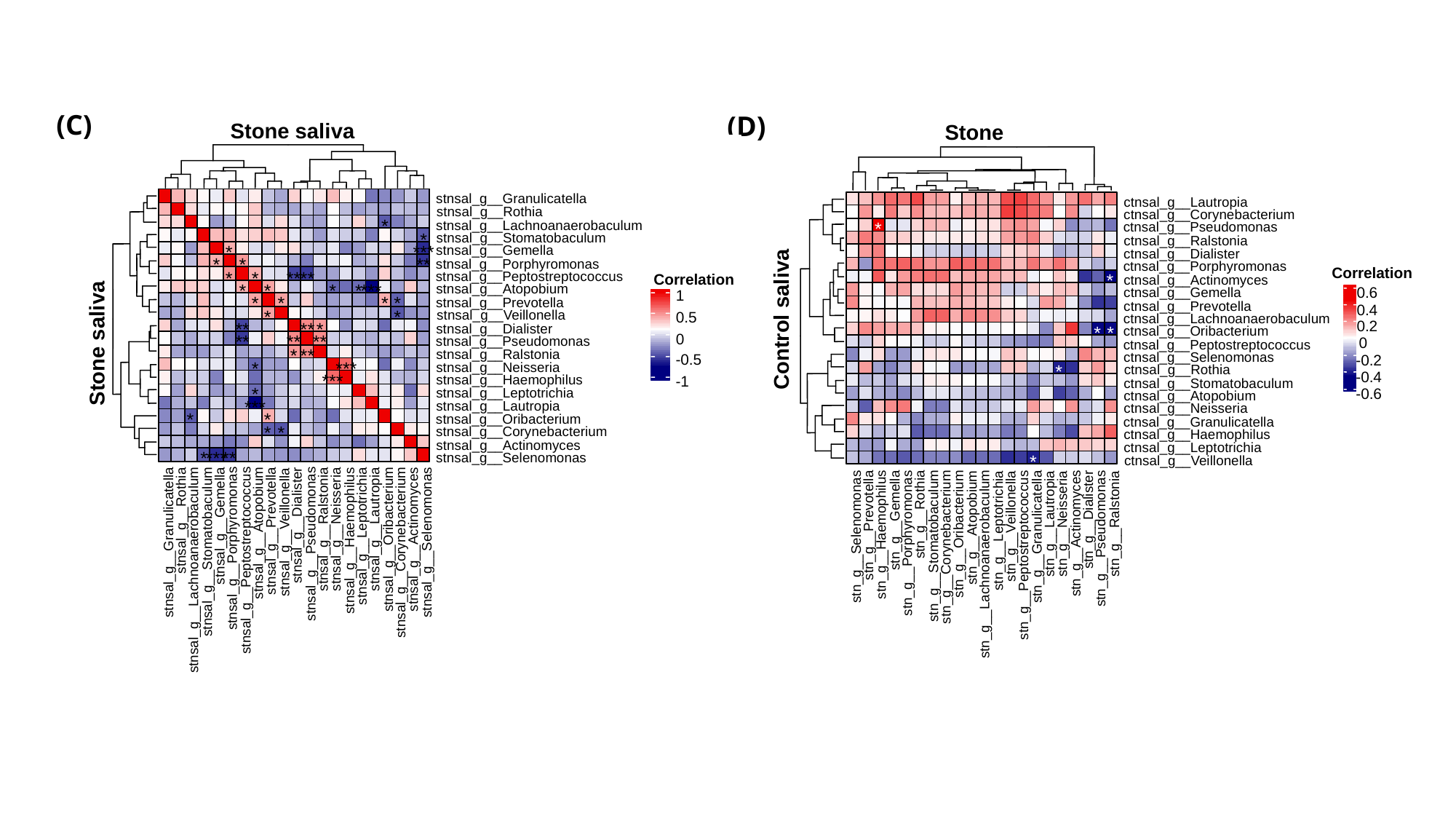

(C)
(D)
Stone saliva
stnsal_g__Granulicatella
stnsal_g__Rothia
stnsal_g__Lachnoanaerobaculum
stnsal_g__Stomatobaculum
stnsal_g__Gemella
stnsal_g__Porphyromonas
stnsal_g__Peptostreptococcus
Correlation
stnsal_g__Atopobium
1
stnsal_g__Prevotella
stnsal_g__Veillonella
0.5
stnsal_g__Dialister
0
stnsal_g__Pseudomonas
stnsal_g__Ralstonia
-0.5
stnsal_g__Neisseria
stnsal_g__Haemophilus
-1
stnsal_g__Leptotrichia
stnsal_g__Lautropia
stnsal_g__Oribacterium
stnsal_g__Corynebacterium
stnsal_g__Actinomyces
stnsal_g__Selenomonas
stnsal_g__Rothia
stnsal_g__Dialister
stnsal_g__Gemella
stnsal_g__Ralstonia
stnsal_g__Neisseria
stnsal_g__Lautropia
stnsal_g__Prevotella
stnsal_g__Veillonella
stnsal_g__Atopobium
stnsal_g__Leptotrichia
stnsal_g__Oribacterium
stnsal_g__Actinomyces
stnsal_g__Haemophilus
stnsal_g__Granulicatella
stnsal_g__Selenomonas
stnsal_g__Pseudomonas
stnsal_g__Porphyromonas
stnsal_g__Stomatobaculum
stnsal_g__Corynebacterium
stnsal_g__Peptostreptococcus
stnsal_g__Lachnoanaerobaculum
*
*
*
***
*
*
**
*
*
**
**
*
*
*
*
***
*
*
*
*
*
*
**
**
*
**
**
**
*
**
*
***
***
*
***
*
*
*
*
*
***
**
Stone saliva
Stone
*
*
*
*
*
*
ctnsal_g__Lautropia
ctnsal_g__Corynebacterium
ctnsal_g__Pseudomonas
ctnsal_g__Ralstonia
ctnsal_g__Dialister
ctnsal_g__Porphyromonas
Correlation
0.6
0.4
0.2
0
-0.2
-0.4
-0.6
ctnsal_g__Actinomyces
ctnsal_g__Gemella
ctnsal_g__Prevotella
Control saliva
ctnsal_g__Lachnoanaerobaculum
ctnsal_g__Oribacterium
ctnsal_g__Peptostreptococcus
ctnsal_g__Selenomonas
ctnsal_g__Rothia
ctnsal_g__Stomatobaculum
ctnsal_g__Atopobium
ctnsal_g__Neisseria
ctnsal_g__Granulicatella
ctnsal_g__Haemophilus
ctnsal_g__Leptotrichia
ctnsal_g__Veillonella
stn_g__Rothia
stn_g__Dialister
stn_g__Gemella
stn_g__Lautropia
stn_g__Neisseria
stn_g__Ralstonia
stn_g__Prevotella
stn_g__Veillonella
stn_g__Atopobium
stn_g__Leptotrichia
stn_g__Actinomyces
stn_g__Oribacterium
stn_g__Haemophilus
stn_g__Selenomonas
stn_g__Granulicatella
stn_g__Pseudomonas
stn_g__Porphyromonas
stn_g__Stomatobaculum
stn_g__Corynebacterium
stn_g__Peptostreptococcus
stn_g__Lachnoanaerobaculum

## Slide 9
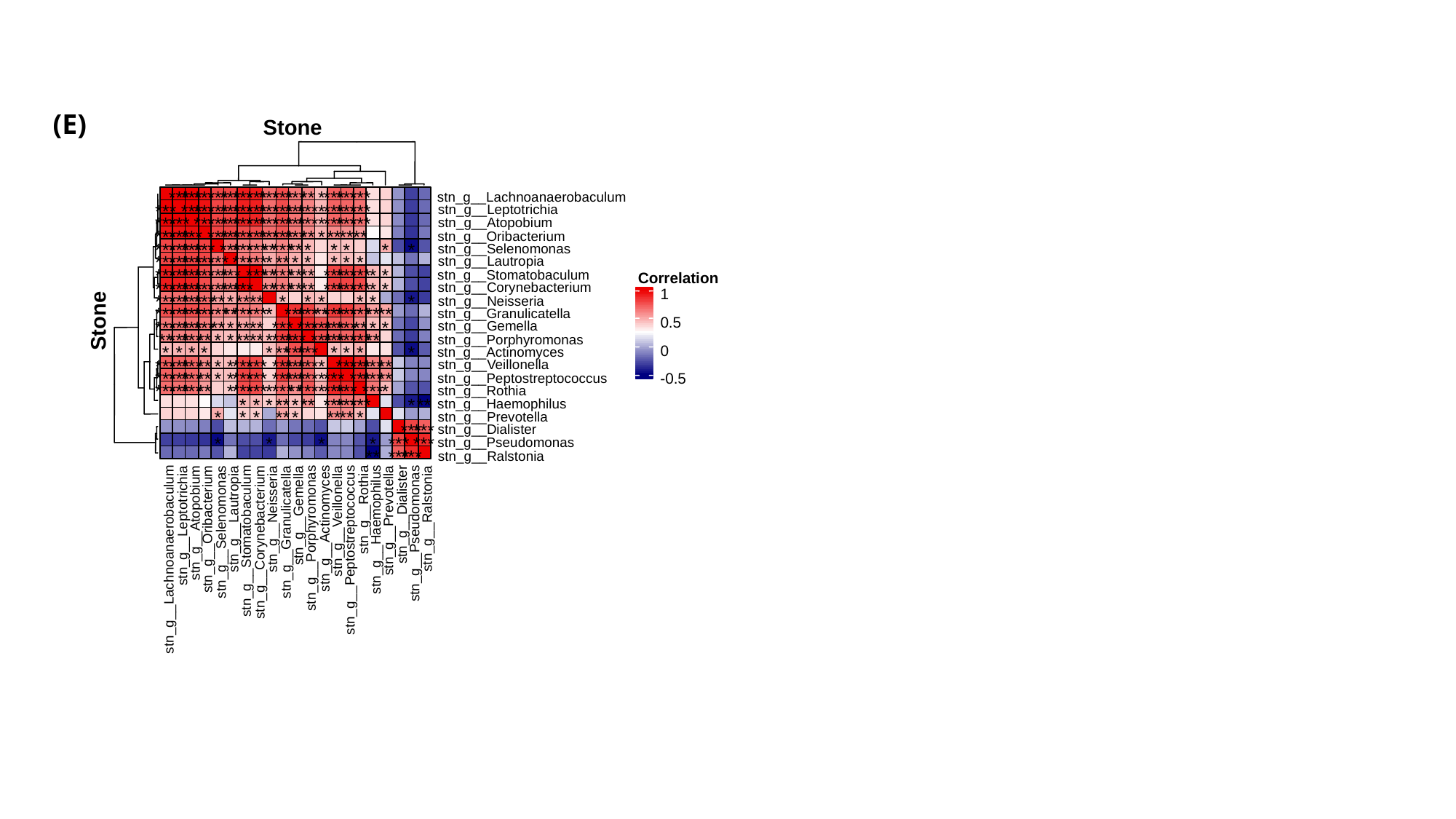

(E)
Stone
stn_g__Lachnoanaerobaculum
stn_g__Leptotrichia
stn_g__Atopobium
stn_g__Oribacterium
stn_g__Selenomonas
stn_g__Lautropia
stn_g__Stomatobaculum
Correlation
stn_g__Corynebacterium
1
stn_g__Neisseria
stn_g__Granulicatella
0.5
stn_g__Gemella
stn_g__Porphyromonas
0
stn_g__Actinomyces
stn_g__Veillonella
-0.5
stn_g__Peptostreptococcus
stn_g__Rothia
stn_g__Haemophilus
stn_g__Prevotella
stn_g__Dialister
stn_g__Pseudomonas
stn_g__Ralstonia
stn_g__Rothia
stn_g__Dialister
stn_g__Gemella
stn_g__Ralstonia
stn_g__Lautropia
stn_g__Neisseria
stn_g__Prevotella
stn_g__Veillonella
stn_g__Atopobium
stn_g__Leptotrichia
stn_g__Actinomyces
stn_g__Oribacterium
stn_g__Haemophilus
stn_g__Selenomonas
stn_g__Granulicatella
stn_g__Pseudomonas
stn_g__Porphyromonas
stn_g__Stomatobaculum
stn_g__Corynebacterium
stn_g__Peptostreptococcus
stn_g__Lachnoanaerobaculum
***
***
***
***
***
***
***
***
***
***
**
*
***
***
***
***
***
***
***
***
***
***
***
***
***
***
*
***
***
***
***
***
***
***
***
***
***
***
***
***
***
*
***
***
***
***
***
***
***
***
***
***
***
***
***
**
*
**
**
**
***
***
***
***
***
***
***
**
***
**
*
*
*
*
*
***
***
***
***
***
***
***
*
**
*
*
*
*
*
***
***
***
***
***
***
***
**
***
**
**
***
***
***
*
*
***
***
***
***
***
***
***
**
***
**
**
***
***
***
*
*
***
***
***
***
**
*
**
**
*
*
*
*
*
*
***
***
***
***
***
**
***
***
*
***
***
**
***
***
***
**
**
***
***
***
***
**
*
**
**
***
***
***
***
***
**
*
*
**
***
***
**
*
*
**
**
*
***
***
***
***
***
***
**
*
*
*
*
*
**
***
***
*
*
*
*
***
***
***
**
*
*
***
***
***
***
***
*
***
***
***
**
***
***
***
**
*
*
***
***
***
***
***
*
***
***
***
**
***
***
***
**
*
***
***
*
***
**
***
*
***
***
***
*
*
*
*
**
*
**
***
***
***
*
**
*
*
*
**
*
**
**
*
***
***
*
*
*
*
***
***
**
***
***
Stone

## Slide 10
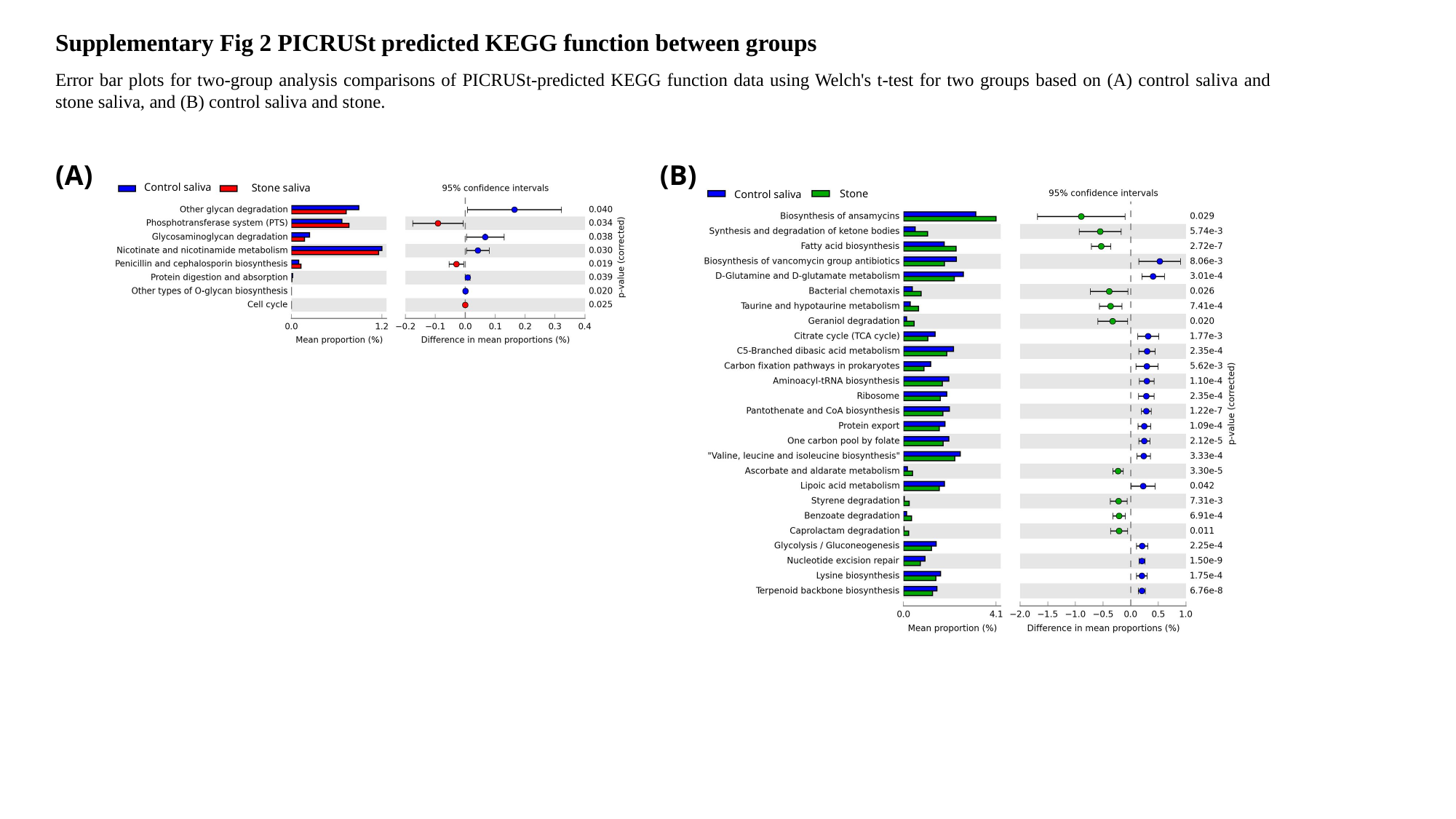

Supplementary Fig 2 PICRUSt predicted KEGG function between groups
Error bar plots for two-group analysis comparisons of PICRUSt-predicted KEGG function data using Welch's t-test for two groups based on (A) control saliva and stone saliva, and (B) control saliva and stone.
(A)
(B)
Control saliva
Stone saliva
Stone
Control saliva
